# Solving the stereo correspondence problem with false matches

**DOI:** 10.1371/journal.pone.0219052

**Published:** 2019-07-29

**Authors:** Cherlyn J. Ng, Bart Farell

**Affiliations:** Institute for Sensory Research, Department of Biomedical and Chemical Engineering, Syracuse University, Syracuse, New York, United States of America; University of British Columbia, CANADA

## Abstract

The stereo correspondence problem exists because false matches between the images from multiple sensors camouflage the true (veridical) matches. True matches are correspondences between image points that have the same generative source; false matches are correspondences between similar image points that have different sources. This problem of selecting true matches among false ones must be overcome by both biological and artificial stereo systems in order for them to be useful depth sensors. The proposed re-examination of this fundamental issue shows that false matches form a symmetrical pattern in the array of all possible matches, with true matches forming the axis of symmetry. The patterning of false matches can therefore be used to locate true matches and derive the depth profile of the surface that gave rise to them. This reverses the traditional strategy, which treats false matches as noise. The new approach is particularly well-suited to extract the 3-D locations and shapes of camouflaged surfaces and to work in scenes characterized by high degrees of clutter. We demonstrate that the symmetry of false-match signals can be exploited to identify surfaces in random-dot stereograms. This strategy permits novel depth computations for target detection, localization, and identification by machine-vision systems, accounts for physiological and psychophysical findings that are otherwise puzzling and makes possible new ways for combining stereo and motion signals.

## Introduction

A necessary step in the stereoscopic reconstruction of 3-D surfaces is to select correspondences between image features captured by multiple cameras or eyes. To usefully combine the two images (‘L’ and ‘R’) and extract depth information, the system must distinguish ‘true matches’, which combine L- and R-image elements whose source is a single environmental feature, and ‘false matches’, which combine similar image elements from different objects or from different parts of the same object. False matches create the correspondence problem; they mimic true matches while specifying disparities between the two images other than those produced by real objects.

Traditional solutions to the correspondence problem start with constraints consistent with natural image properties. These constraints are used to reinforce likely true matches and suppress likely false ones. Often this is done through refinements and elaborations of strategies set out in Julesz’s [1] and Marr and Poggio’s [2,3] cooperative algorithms, which continue to stimulate developments in psychophysical, physiological, and computational approaches to stereoscopic processing, and remain essential components of current, state-of-the-art machine vision methods, e.g., [4–6], also see [7]. These approaches are conventional in that they employ ‘direct’ algorithmic searches; they attempt to find true matches by selecting for likely true matches. The approach considered here is different in kind—an ‘indirect’ approach that locates true matches using cues possessed solely by false matches. It is compatible with both correlation- and feature-based methods of stereo matching, yet dispenses with an integral component of direct approaches, the need to suppress false matches.

**Fig 1:**
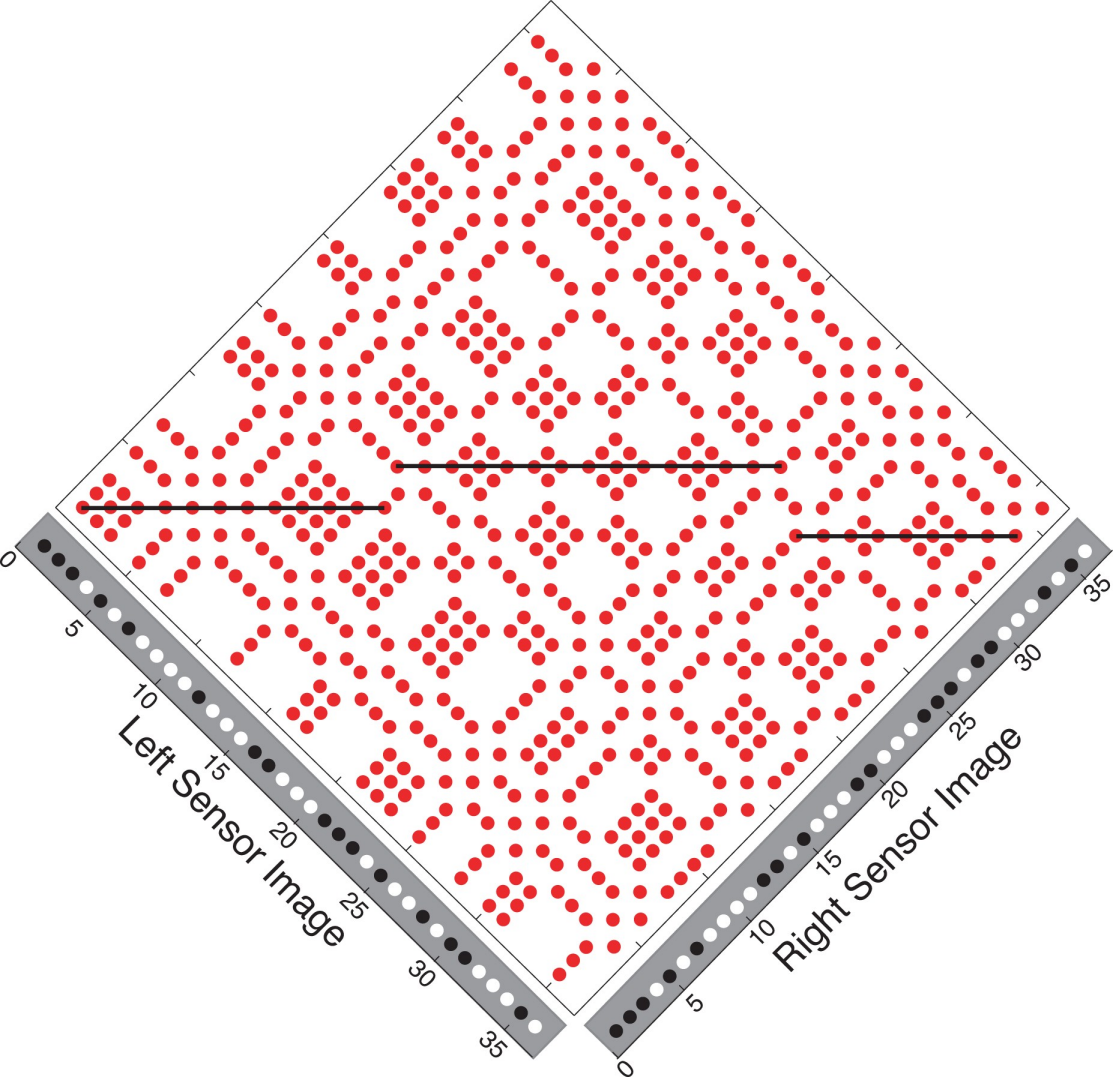
Keplerian array with true matches linked by horizontal lines. L and R images are presented in the margins and compared in an orthogonal array space. Points in the L image that match points in the R image (black/black or white/white combinations) are denoted by red dots in the array, while mismatching points (white/black combinations) are left blank. The orthogonal format of this and subsequent depictions of the KA has been adopted for convenience. A more realistic array, one reflecting actual sensor positions, could have been used but to no advantage. The transformation to and from the orthogonal format is straightforward and bestows both conceptual and computational benefits.

The full set of matches, both true and false, is expressed in the Keplerian array (KA). Fig 1 shows a KA produced by two 1-dimensional binary random-dot images, which appear on the margins. The KA compares the features of the two images and classifies each feature pair as matching (red dot) or mismatching (white). The image features themselves can be defined as desired. The x-direction of visual space is coded along the horizontal direction within the KA and the z-direction is coded along the vertical direction. There are many more matches than image dots. Each black or white dot produces multiple matches, only a small fraction of which are true matches. Horizontal lines in Fig 1 connect the true matches produced by three frontoparallel surfaces; all the other matches are false matches. The problem of winnowing true from false matches—the correspondence problem—can be especially challenging when target objects are presented under camouflaged or crowded conditions.

**Fig 2:**
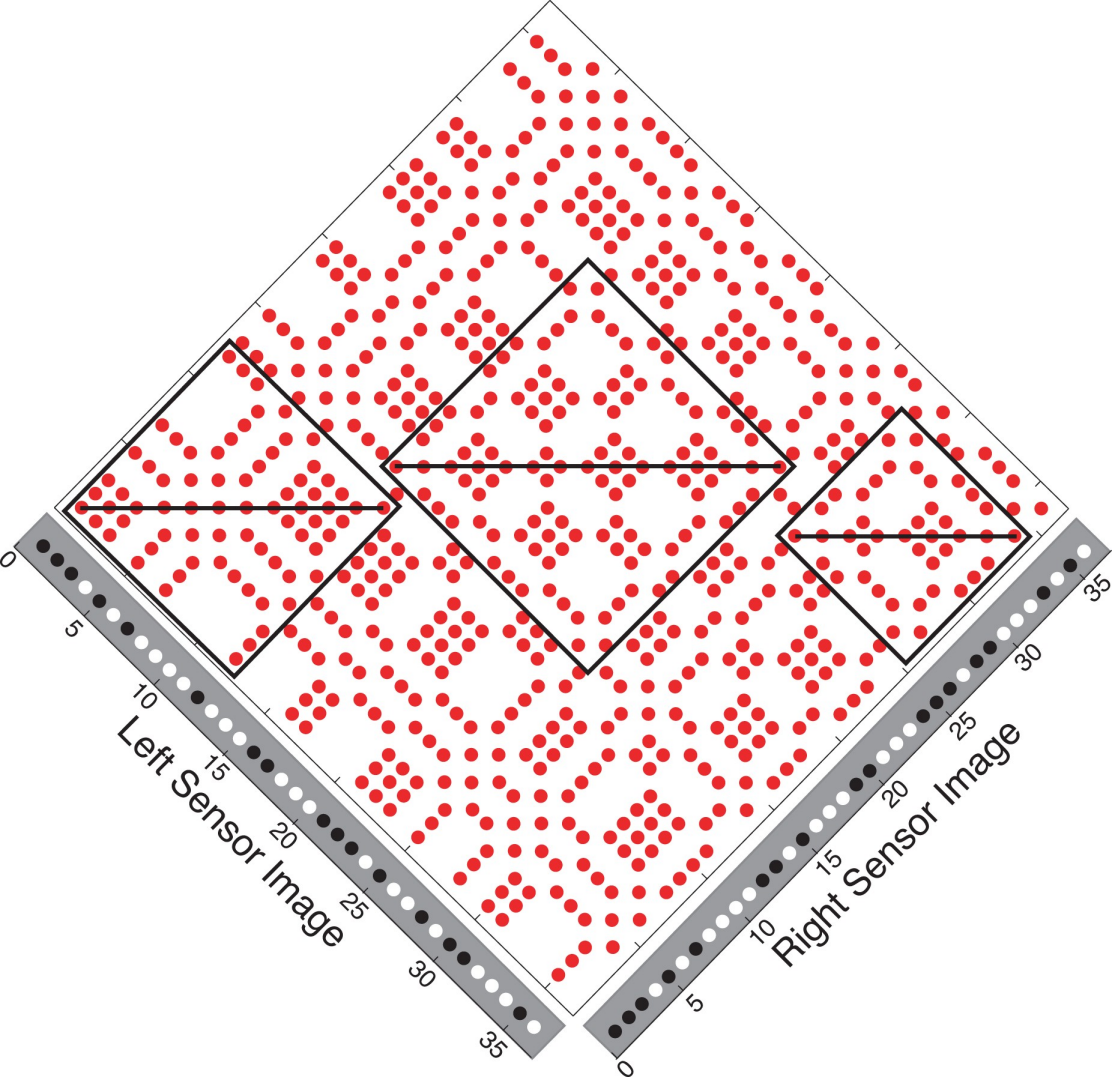
False match symmetry zones. False matches form diamond-shaped symmetry zones (boxed regions) surrounding the true matches of frontoparallel surfaces. The true matches lie along the axis of symmetry.

**Fig 3:**
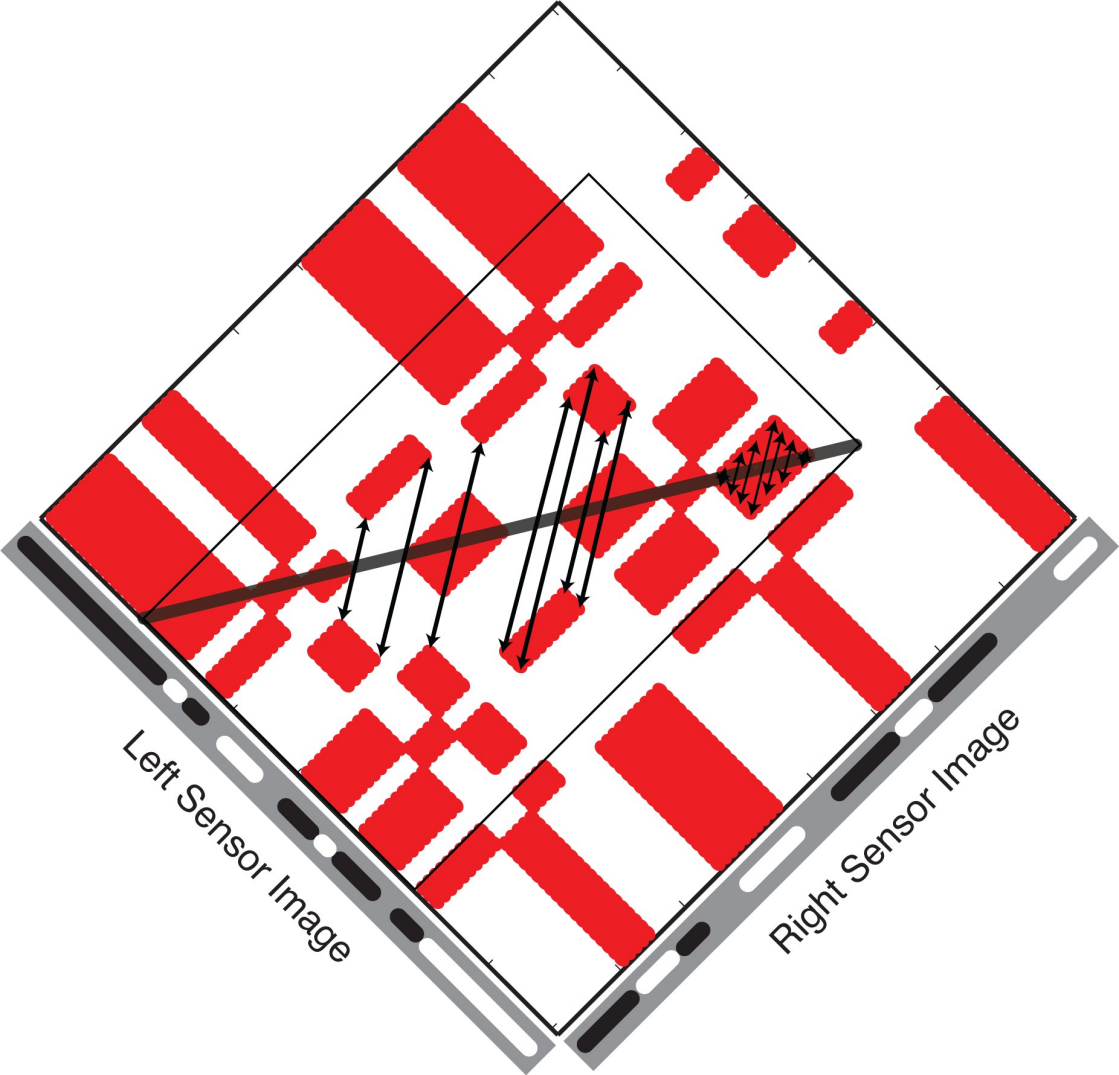
The sheared symmetry zone surrounding the true matches of a slanted planar surface. A bold black line has been drawn through the true matches generated by a slanted planar surface. Red regions off this line are false matches. The thin lines form a rectangle bounding the region of false-match symmetry. Discrete matches, as shown in Figs 1 and 2, have been merged into continuous blocks to emphasize their shapes and sizes. Arrows link symmetrical false matching nodes.

**Fig 4:**
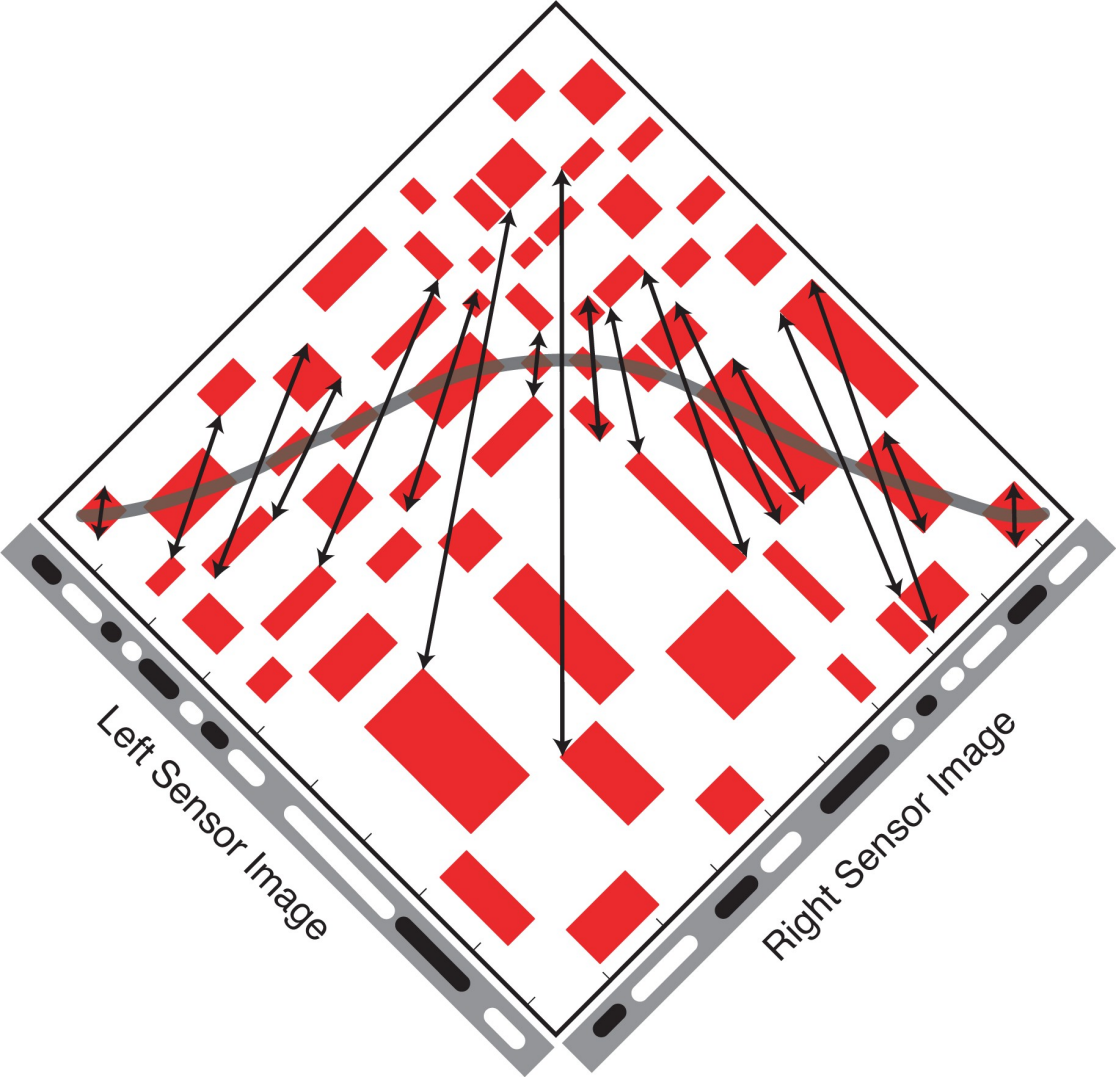
Surface curvature smoothly varies the shearing transformation of the false-match symmetry. A curved surface is composed of local planar segments defined by contiguous points of identical texture elements. The result is a continuous variation in the difference between aspect ratios of corresponding matching blocks. Corresponding points on these blocks are linked here by arrows.

We show here that instead of being a source of noise, false matches hold information about the location of true matches. True matches can be thought of as an axis of symmetry in the Keplerian array. False matches are symmetrically distributed on either side of this axis. The two sides are related to the other by a scaling transformation that specifies the depth profile of the surface generating the true matches. Fig 2 shows an example for the case of frontoparallel surfaces, which produce a reflection symmetry within the diamond-shaped symmetry zones in the orthogonal arrays used here. Surface slant adds an additional differential scaling transformation to the symmetry (Fig 3; arrows link symmetrical false-match nodes). This changes the aspect ratio of matching blocks across the symmetry axis and hence changes the aspect ratio of the symmetry region itself. For planar surfaces, aspect ratios of corresponding matching blocks differ by a constant ratio that is directly related to surface slant. For curved surfaces (Fig 4), this ratio is no longer constant. Instead, it undergoes a systematic variation across contiguous corresponding matching blocks. This variation depends directly on the curvature. Depending on the curvature parameters, this introduce a second-order expansion/contraction transformation of corresponding matching blocks across the symmetry axis.

Thus, each set of potential true matches—each contiguous string of nodes in Figs 2, 3 and 4 with a monotonic ordering along the x-axis (horizontal)—is flanked by arrays of nominally false matches. The symmetry function relating the two flanking regions describes the depth profile of the true-match surface. This function ranges from a simple reflection of matching regions across a frontoparallel symmetry axis to a haphazard pairing across a random depth profile, which is the ‘symmetry axis’ created by uncorrelated L and R input. The symmetry parameters form an alternative coding of true-match structure, no less accurate for the symmetrical false matches being ‘non-veridical’.

The sources of the true matches in Figs 1–4 are perfectly camouflaged surfaces (see the marginals of the KAs); there are no signals corresponding to the surfaces in either monocular image alone, only in the correlation between the images. Outside these symmetry zones, the expected patterning is random.

For frontoparallel surfaces the reason for the symmetry is this: Let surface image points *L(i)* and *R(j)* on the left and right retinas be true matches. Then, for unequal integers *m* and *n*, if surface image points *L(i+m)* and *R(j+n)* falsely match, then so do *L(i+n)* and *R(j+m)* (because in order for the surface to be frontoparallel, *L* values must equal *R* values within the limits of the surface’s coordinates). A non-frontoparallel surface warps this reflection correspondence between the *L(i+m)*:*R(j+n)* and *L(i+n)*:*R(j+m)* pairs, for there is no longer equality (with disparity offset) between *L* and *R*. Yet the inequality—the disparity function that relates the left and right retinal images—is still encoded in the false matches; the warping fully preserves the surface’s deviation from the frontoparallel plane.

In an antecedent of our approach, Tyler [8–9] observed that a surface on the horopter (i.e., having a disparity of zero) divides the KA into two sets of ‘conjugate pairs’, false matches having the same disparity magnitude but opposite signs. He suggested disparity averaging along lines of sight as a strategy for cancelling the conjugate pairs and thereby exposing the true matches. He also pointed out a limitation of this strategy: Conjugate false matches for slanted surfaces are not arrayed along lines of sight and therefore are not cancelled by averaging. Similarly, averaging fails for frontoparallel surfaces that do not lie on the horopter and for those that have extents less than the full KA. In both of these cases, conjugate pairs do not extend in depth across the entire KA; therefore, averaging along lines of sight does not eliminate all but true-match disparity values. However, false-match symmetry can be put to use in ways other than averaging, which we demonstrate here.

While false-match symmetry can be used to resolve the correspondence problem, it is not sufficient for the perception of depth in biological—at least primate—visual systems. Interocularly anticorrelated stimuli and their correlated counterparts produced KAs that are in anti-phase: matches replace mismatches and *vice versa*. As a result, the two stimulus types produce the same symmetry-zone parameters. However, anticorrelated stimuli lead to mismatches instead of matches along the axis of symmetry and generally do not lead to the perception of depth [10,11]. Thus, for animal vision false-match symmetry must be regarded as a cue to the location of true matches rather than a direct signal of surface depth.

### Using false-match symmetry to sense surface shape

The three classes of surface shape in depth—frontoparallel surfaces, slanted planar surfaces, and curved surfaces—each generates false-match symmetry zones in the KA that are distinguished by the transformation relating the false matches on one side of the symmetry axis to the corresponding false matches on the other side. We use random-dot stereograms (RDSs) to show the feasibility of recovering the depth profile of these surfaces using false-match symmetry. We also examine the effects of false-match probability and luminance differences between the L and R images, which mimic the influence of noise or a non-Lambertian reflectance function. Our goal is to show that false-match symmetry can be used to recover surface depth. There are many potential algorithms for detecting false-match symmetry, including hybrid approaches in combination with direct searches for true matches. Ours was designed to allow us to study intermediate computational states, not to optimize performance for a particular criterion. The method we use seeks, in effect, to characterize all possible symmetry regions in the KA and to select those that correspond to the symmetrical false matches likely generated by smooth depth profiles. The method assumes that surfaces are larger than clusters of texture elements having similar statistics; it assumes, for example, that the binary colored surface is not all white or all black. Because the texture elements are randomly colored, this assumption is, of course, probabilistic.

## Method

### Stimuli

In order to simulate the highest degree of camouflage, we generated instances of pairs of one-dimensional random-dot images that had equal proportion of randomly assigned white and black dots. Each image was composed of 1x101 pixels. There were no monocular cues to the position or the shape of the surface, which was defined by disparity only and revealed by combining the two images into a RDS. The RDSs contained camouflaged surfaces whose depth profiles were either frontoparallel, slanted, or curved and whose extent spanned all or part of the 101 pixels of the stereogram. We also generated stereograms with grayscale intensities by randomly assigning each pixel with a floating-point number between zero to one from a uniform distribution. These also depicted frontoparallel, slanted, or curved surfaces, but generated fewer false matches than the binary images.

A set of conditions introduced noise into the pixel intensities to simulate non-Lambertian reflections and other factors that render corresponding points unequal in intensity. We varied both the proportion of pixels that carried noise and the amount of noise between corresponding points. Noise was randomly selected from a uniform distribution up to the maximum allowable intensity difference, halved, and applied to the L and R images with opposite polarities (noise intensity subtracted from the R image if added to the L, and vice versa).

### Image processing and constructing Keplerian arrays

The KAs shown in Figs 1–4 were formed by simple arithmetical combination of L and R image elements. The false-match symmetry parameters within such KAs could be extracted directly in order to locate the symmetry axis and identify candidate true matches. However, in physically realized systems, non-Lambertian reflectances, pre-processing operations, and noise would make their own contributions. The resulting KA would differ from the idealized arithmetical KA and would be more informative from an implementational view. We chose to construct the KA by optionally adding intensity noise, filtering each image with an array of paired L and R Gabor kernels, and combining the filtered output into Keplerian arrays whose false-match symmetry could be scored using these filtered outputs to identify regions of symmetry and candidate true matches.

**Fig 5:**
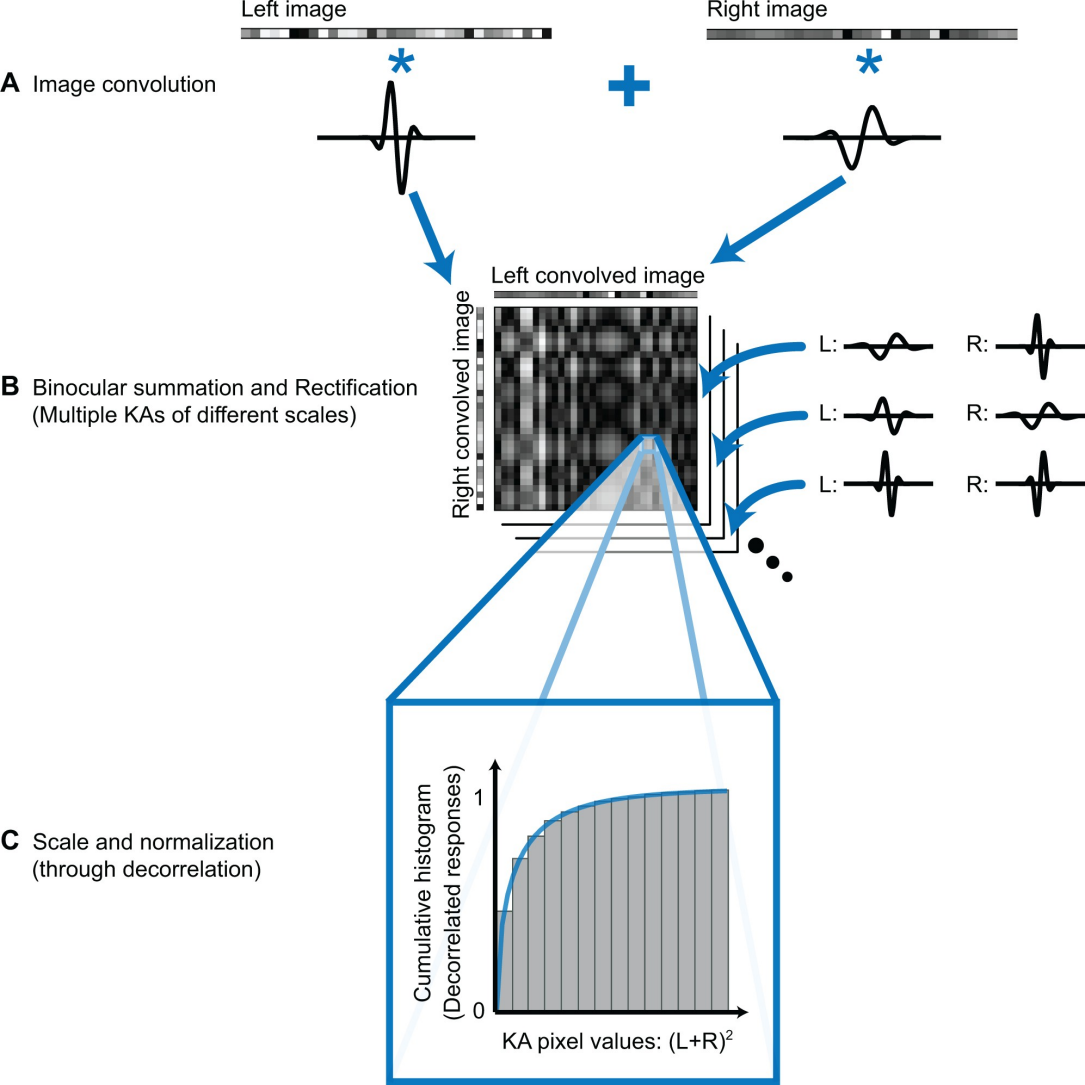
Reconstruction of depth profiles using false match symmetry in Keplerian arrays. (A) Stereo images were first convolved with odd-symmetric Gabor kernels of various sizes. (B) The convolution products of each KA were summed and rectified (squared) at every possible pixel combination. White patches indicate highly symmetrical, and black patches, unsymmetrical. (C) Decorrelation scaled and normalized the rectified binocular signals according to its cumulative distribution so that KAs sampled at different resolutions are comparable (see Supplementary Methods in S1 Text).

In order to visualize KAs and identify symmetrical patterns at different spatial resolutions, images were convolved with pairs of antiphase odd-symmetric Gabor kernels of various sizes (Fig 5A). Kernel size was varied by incrementing the wavelength of grating component in steps of single pixels, while keeping the standard deviation of the Gaussian envelope always at 4⨉ the wavelength. This was a monocular operation. Products from every L kernel at the various resolutions were then additively combined with the products of every antiphase R kernel of various resolutions. This created multiple KAs; there were *n*×*m* KAs, given *m* differently sized L kernels and *n* differently sized R kernels (Fig 5B). Paired L and R kernels were in antiphase in order to pick up false matches, as explained below.

### Match determination and pooling

Matches within a KA were determined pixelwise, by summing the left and right convolution products. The combined convolution products define a potential symmetry zone. True matches lie along the zero crossing of the kernels and so would cancel. False matches flanking the symmetry axis and parallel to the other diagonal would have identical but oppositely signed outcomes (Figure B in S1 Text). This false-match outcome was used as evidence of symmetry. Thus, each KA produced by the steps above will have a different spatial resolution, where each pixel in the KA contained a scaled and normalized signal that represented the degree to which L and R images matched at the positions corresponding to that pixel (Fig 5C; see S1 Text for details).

**Fig 6:**
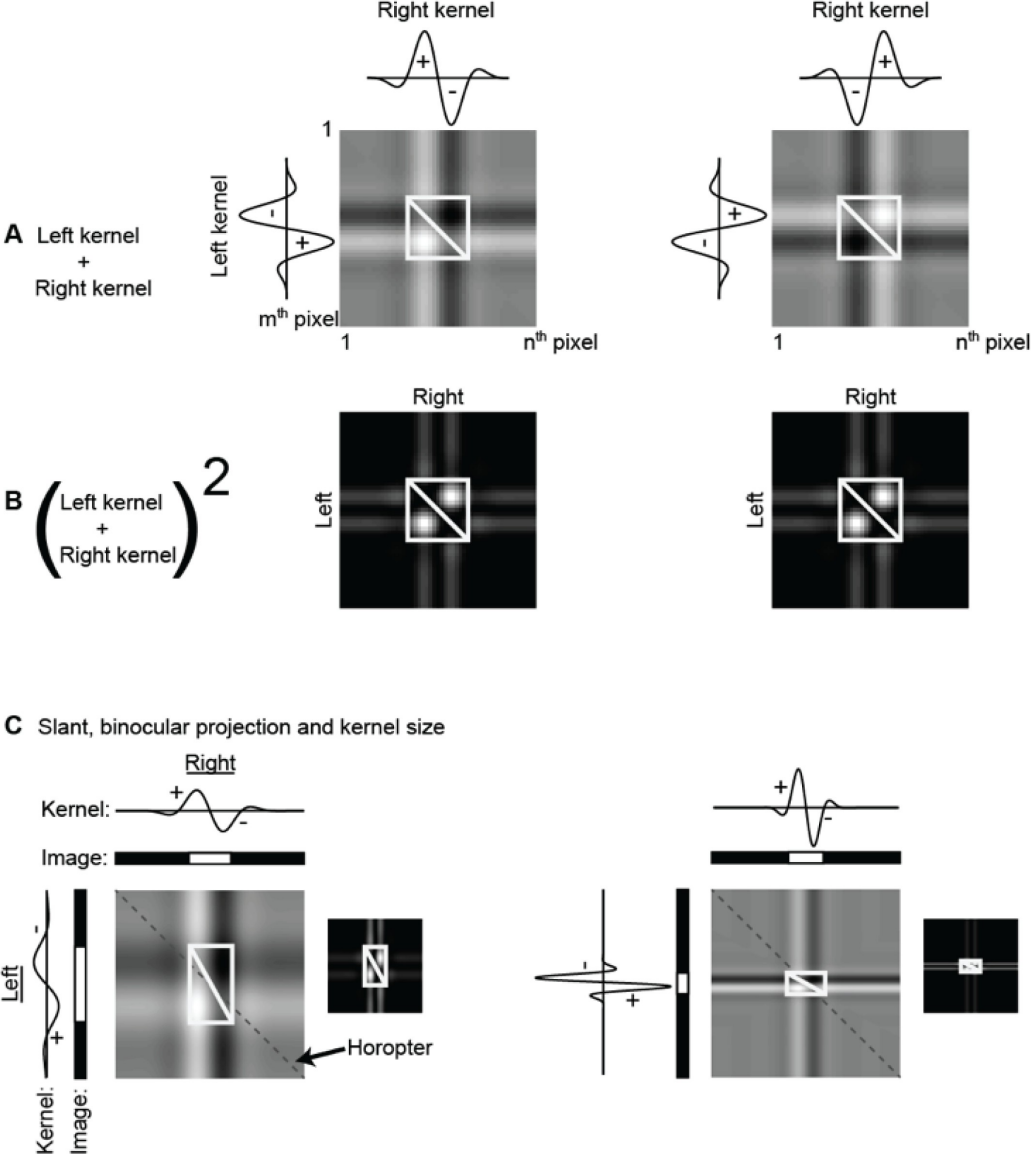
Binocular receptive fields obtained by summing and squaring phase-reversed monocular receptive fields. (A) Two examples of L and R antiphase kernels. Kernels pairs include those with the same and different sizes. Their summation results in odd-symmetric binocular receptive fields where the negative (dark) and positive (light) regions lie on either side of the symmetry zone (white box) flanking the true matches (white diagonal). Near-zero sums (A) are indicative of true match symmetry axes, and rectification (B) would give positive outcomes in all other cases. (C) Two examples in which the surface is a slanted plane of different sizes. Insets show the rectified binocular receptive fields.

The coarsest resolution at which symmetry can be detected within such a zone is indicative of the width of the candidate true match, while the ratio of left and right kernel sizes—the aspect ratio of their combination—yielding the strongest evidence of symmetry indicates its slant (Fig 6; also see Figure B in S1 Text). To accumulate the evidence for symmetry across spatial resolutions, the signals within each KA were first rectified by squaring and then pooled and ranked across KAs of different kernel resolutions and L and R aspect ratios (see Figure C in S1 Text). The false-match symmetry that flanks true matches should be consistent across all resolutions within the symmetry zone and will reinforce rather than cancel when combined.

### Response optimization

Image processing was initialized with Gabor kernels with standard deviations that ranged from a third of the image resolution to a third of the Nyquist frequency. This range was chosen to enable 99% of the information contained within 2 pixels to be sampled by the smallest kernel, and 99% of the entire image to be sampled by the largest kernel. Threshold was initially set at the 95th percentile. These initial parameters may not be optimum for the surface properties in the stereogram (see Results). Hence, the range of the Gabor kernels and the threshold were iteratively optimized from the initial estimate by an unsupervised combination of simulated annealing and least squares minimization to maximize the signal-to-noise ratio.

The objective function maximized the signal-to-noise ratio, which in this implementation was defined as evidence for the presence of slants in the individual channels. Accordingly, the averaged data in each slant’s channel were subjected to the Hough Transform. The signal-to-noise ratio in each channel was:

$$SNR=\frac{var\;\left(\theta \right)}{var\;\left(\theta \;\notin \;Pop\right)}$$

where *θ* is orthogonal to the expected slant angle in each channel and *Pop* represents all angles in the transform.

S1 Fig gives the step-by-step procedure of the methods using an example random-dot stereogram as input.

## Results

Our technique selects solutions by identifying symmetrical regions of false matches in Keplerian arrays (Fig 5). The size of the symmetrical region is correlated with the extent of the target surface, while the aspect ratio of the symmetrical region is related to surface slant (Figs 2 and 3). For each surface type, the images we used for analysis were one-dimensional horizontal strips taken from each pair of twenty 101x101-pixel RDSs. Pixels in these RDSs were had either binary intensity values or were grayscale, with randomly assigned intensity values taken from a uniform distribution between 0 and 1. Each image pair was convolved with odd-symmetric Gabor kernels of different sizes. The kernels for L and R images were in anti-phase. Examples of a single pair of kernels applied to the two images are shown in the marginals of Fig 6A. Matches between L and R images were made by summing pairs of filter outputs. The output of filters of all combinations of sizes were summed, each combination producing a KA. Summation of L and R filter output is equivalent to placing an odd-symmetric binocular receptive field at a depth plane specified by the disparity of the individual positions of the kernels (Fig 6A). A near-zero binocular sum would be consistent with the presence of a surface with that disparity, for the product of one side of the false matches would cancel the product of its symmetrical other side. Asymmetrical regions would produce non-zero signals. Rectification (through squaring) before summation doubles the symmetry scores (Fig 6B). Applying both operations in sequence improves selectivity for true matches over false positives.

Large surfaces generate large symmetry regions with correspondingly large sampling bandwidths (Fig 6C). Selection of candidate surface depth profiles was based on signal strength for each aspect ratio up to the limiting kernel size. Note that this detection scheme makes no special provision for curved surfaces, whose signals will be dispersed across aspect ratios. The following sections present examples of this strategy as applied to random-dot surfaces with different depth profiles and examines the effect of noise level on the outcome. Mean parameter values (kernel sizes, thresholds and mean signal-to-noise ratios) for reconstructed surfaces in a large representative sample of all conditions tested appear in Table 1 at the end of Results.

### Horopter surfaces

**Fig 7:**
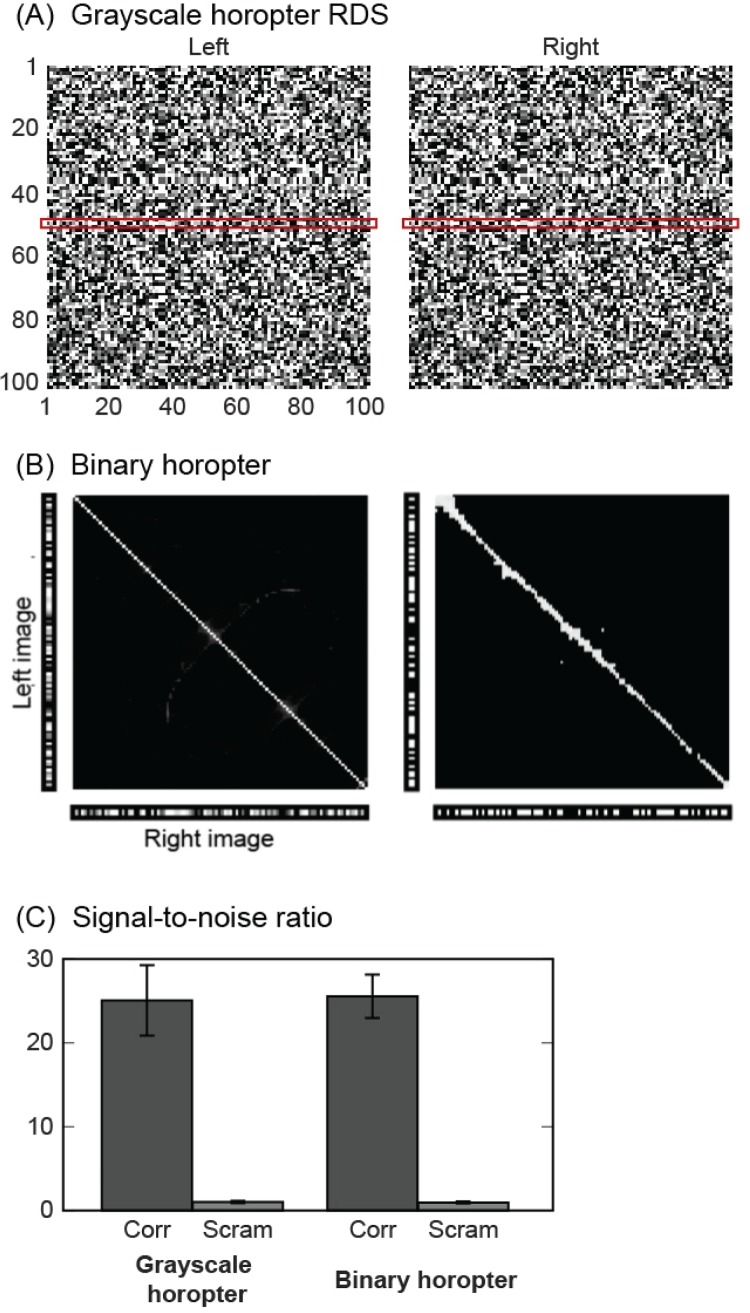
Example RDS and associated depth recovery. (A) An example of a 101x101 grayscale random-dot stereogram. The L and R images are identical. A horizontal row extracted from the middle (red boxes) was provided to the algorithm. (B) Left: True matches fall on the horopter. Right: Surface recovery from an example stereogram. Ground truth to these solutions appears in panel A of S2 Fig. (C) Mean signal-to-noise ratios across 20 simulations each of grayscale and binary stereograms. Error bars indicate standard deviations of the SNR. ‘Corr’ denotes fully correlated RDSs; ‘Scram’ denotes pixel-scrambled controls.

The simplest stereogram consisted to identical grayscale L and R images (Fig 7A), from which a single row (marked by red box) was analyzed. This RDS contains true matches along the horopter spanning the entire image width and conforms to Tyler’s [8,9] conjugate-pair assumptions.

Twenty independent samples of the horopter depth plane were analyzed, each with a randomly selected set of grayscale pixel intensity values. To maximize the number of false matches, we generated another twenty random dot stereograms, now with binary intensity values. Fig 7B shows the reconstructed profiles for these grayscale and binary images. They were supported by L/R kernels with ratios of 1. The Gaussian component of these kernels ranged between 0.1 and 19 pixels (standard deviation of the Gaussian envelope). Including kernel sizes outside this range reduced the signal to noise ratio and increased the number of spurious matches. The means and standard deviations of the SNRs are given in Fig 7C. These SNRs were compared to uncorrelated random dot stereograms control conditions generated by scrambling the pixel intensity values in both L and R images. SNRs for the horopter-surface stereograms were significantly higher than for the uncorrelated stereograms (*p*<0.01; *t*-test) across the 20 samples.

The average performance across the twenty binary stereograms, as measured by the SNR, was no higher than the grayscale stereograms with fewer false matches (Fig 7C), suggesting that though the reconstructions were visibly different, performance had plateaued with respect to the number of false matches.

### Complex surfaces

**Fig 8:**
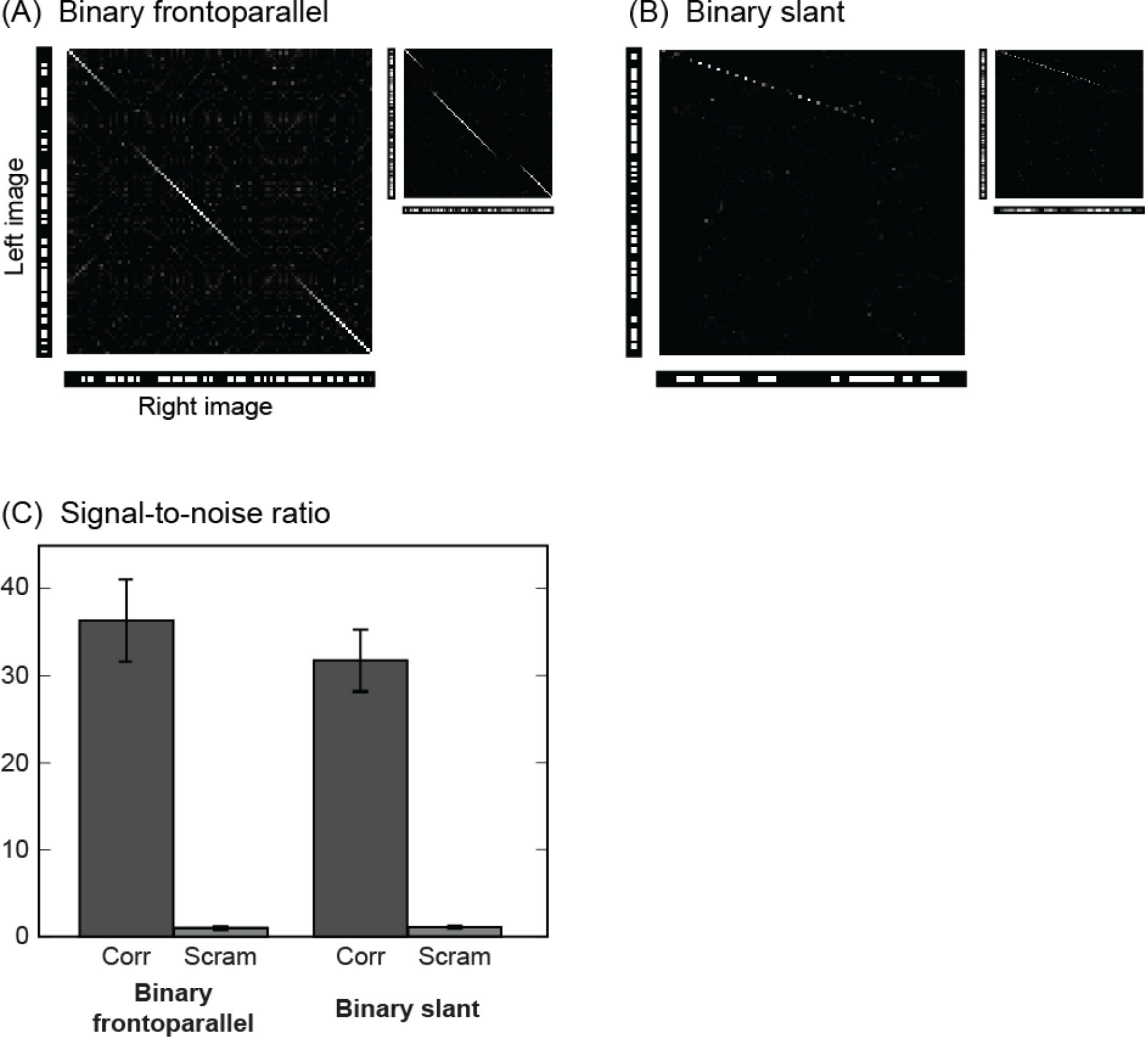
Examples of reconstructed profiles. Surface reconstruction in (A) is from multiple frontoparallel depth planes (ground truth in panel B of S2 Fig) and in (B) from a single 26.5° slanted surface (ground truth in panel C of S2 Fig). Image intensity values were binary (grayscale for insets). (C) Performance with binary and grayscale images for the two surface profiles.

**Fig 9:**
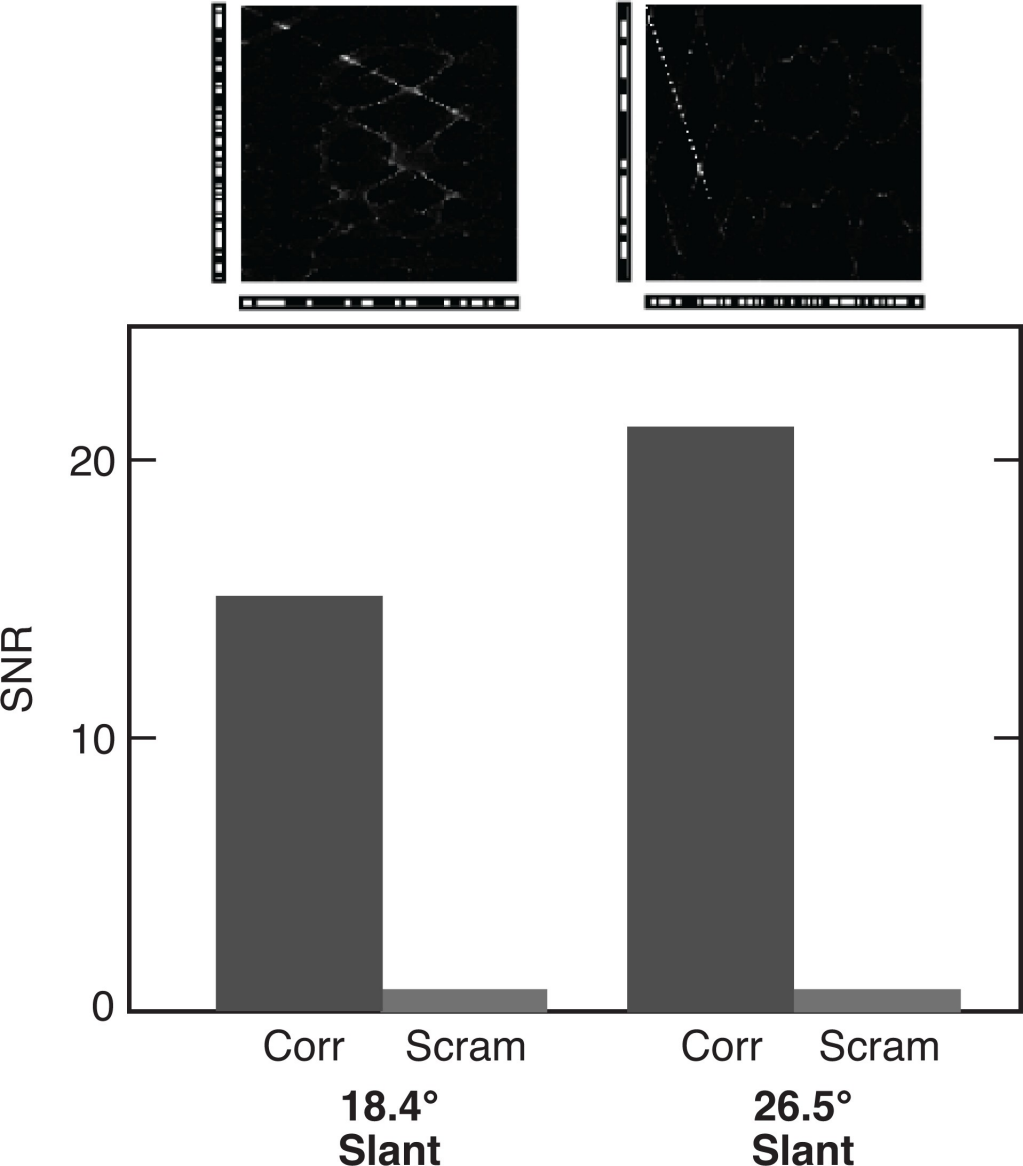
Reconstruction of planar surfaces with different slants. Reconstructions (top) have slants with L and R components in ratios of 2:1 and 3:1. SNRs for each slant (bottom) are compared with scrambled versions of the same stereograms. Ground truths are shown in panels D and E of S2 Fig.

We presented the algorithm with both binary and grayscale stereograms containing three disjoint frontoparallel surfaces, surfaces slanted 18.4° and 26.5° relative to the horopter, and concave curved surfaces. Again, there twenty samples of each case. For frontoparallel and slanted surfaces, the reconstructed depth profiles were similar for binary stereograms (examples of which are shown in Fig 8A and 8B, respectively), and grayscale stereograms (Fig 8A and 8B, insets), showing that performance does not degrade as the number of false matches increases, as it tends to do in other methods. Reconstruction parameters are related to surface size, kernels for the frontoparallel surfaces averaging between 0.46 to 6.8 pixels, with those for the slant averaging between 1.88 to 19.2 pixels. Planar surfaces, regardless of the slant (up to 26.5°), were detected well in RDSs (Fig 9).

Curved surfaces, by contrast, were detected at a low level. The SNR was 1.42. As previously noted, our surface selection procedure favored planar surfaces; moreover, curvature reconstruction is intrinsically more difficult. Spatially asymmetric kernels, which were not utilized here, could prove helpful. SNRs for the depth profiles are given in Fig 8C.

### Added noise

Natural surfaces seldom have Lambertian reflectances. Hence, under natural viewing conditions, corresponding points often differ in intensity. To examine the effect of differences between the luminance of corresponding dots, we introduced random noise into RDSs depicting three frontoparallel surfaces. Noise was manipulated by varying the range of luminance variation across all corresponding dots in the images or by varying the number of noisy corresponding pixels. In the former case, the magnitude of variation was chosen randomly from a uniform distribution from zero to the cutoff. In the latter case, the intensity cutoff was 5%.

**Fig 10:**
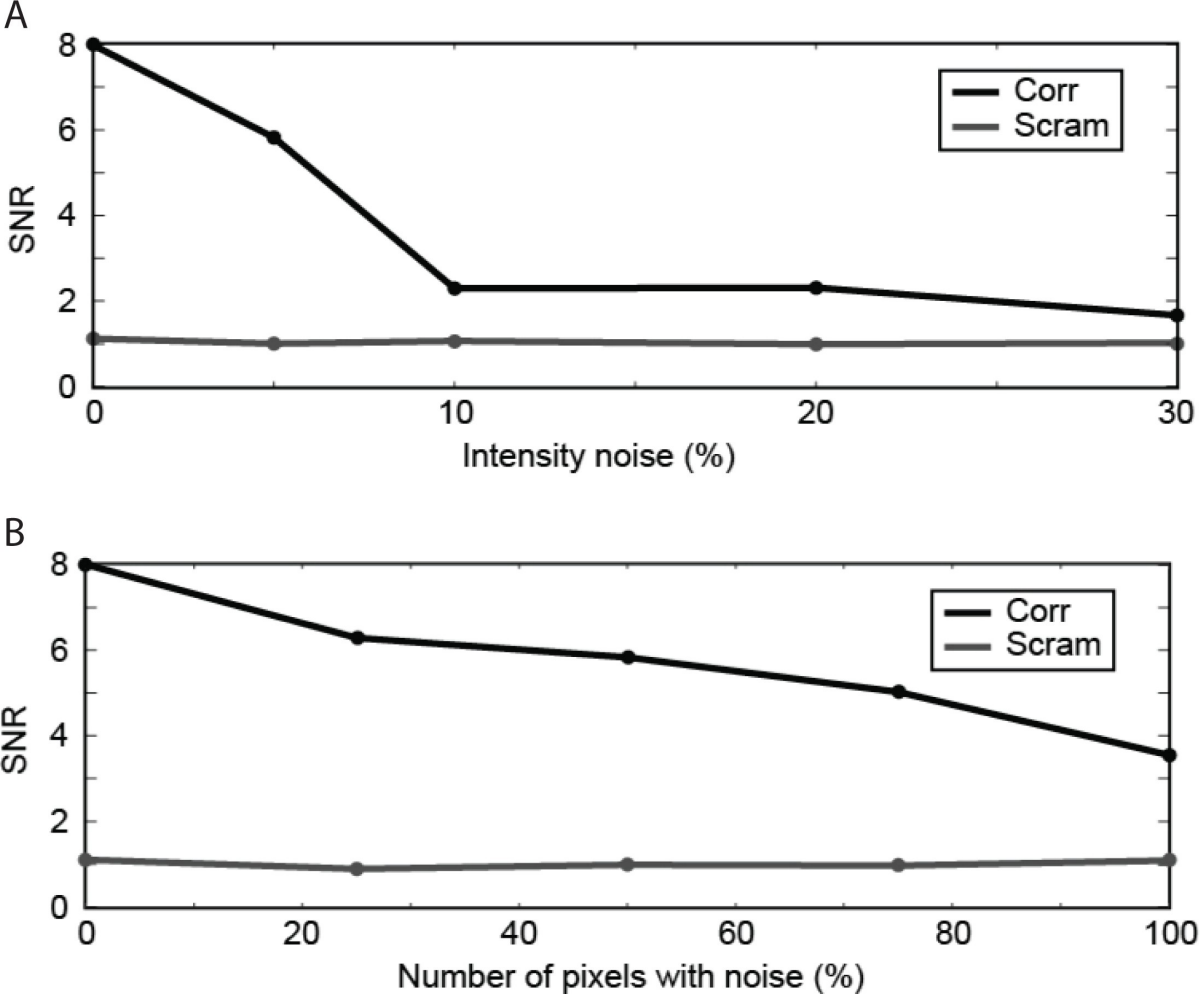
Effect of added intensity noise. (A) Signal-to-noise ratio as a function of corresponding pixel intensity differences. (B) Signal-to-noise ratio as a function of the percentage of noisy pixels. Graphical depictions of the solutions are in S3 Fig. Ground truth to the solutions are in panel D of S2 Fig.

Signal strength dropped sharply as the maximum noise value increased to 10% (Fig 10A). With maximum noise at 30%, the signal was still above chance level, but barely. By contrast, smaller intensity differences were reasonably well tolerated, with SNR gradually declining with the proportion of noisy pixels (Fig 10B). Thus, noiseless images are not necessary for recovery of object surfaces in RDSs by this method. Modifying the algorithm at the filter level to down-weight the contributions of fine-scale kernels might increase noise tolerance generally.

### Results summary

Table 1 gives mean parameter values (kernel sizes, thresholds and mean signal-to-noise ratios) for the reconstructed surfaces in a representative sample of all the planar-surface conditions tested. In all of these cases the symmetry-identified true matches superimposed with ground truth, with minor exceptions that appear at the ends of surfaces in low-signal-to-noise-ratio conditions (see S2 and S3 Figs).

**Table 1:** Results summary. Kernel sizes, threshold values, and mean signal-to-noise ratios for a set of conditions discussed in the text.

Stereogram	Kernel size	Threshold	SNR
Grayscale horopter	0.1–19	0.0001	25.07±8.40SD
Binary horopter	0.1–19		25.58±5.16SD
Binary multi-frontoparallel	0.1–6.5		7.98
Grayscale multi-frontoparallel	0.1–6.5		10.74
Binary 18.4° forward slant	1–19		15.88
Binary 26.5° forward slant	2–19		28.68
Binary 18.4° backward slant	1–19		11.75
Binary 26.5° backward slant	2–19		22.37
Grayscale 26.5° backward slant	2–19		27.5313
Binary 5% noise, 50% pixels	0.1–6.5		5.82
Binary 10% noise, 50% pixels		0.0002	2.29
Binary 20% noise, 50% pixels			2.31
Binary 30% noise, 50% pixels			1.66
Binary 5% noise, 25% pixels		0.0001	6.27
Binary 5% noise, 75% pixels			5.0
Binary 5% noise, all pixels			3.55

## Discussion

### The relation between true and false matches

The distribution of features within stereo image pairs determines the extent to which false matches will populate the KA. When each feature in one image has only one matching counterpart in the other image, then there are no false matches and no correspondence problem. Stereopsis is then ‘local’ [1]. We show here that if false matches do co-exist with true matches, the organization of the two is tightly coupled. In particular, false matches within the texture of a single surface are reflected across the true matches in the KA (Fig 3; arrows within the red block). Locating this symmetry and characterizing the relationship between the two false-match regions can specify the position and depth profile of object surfaces. Detecting false-match symmetry is a novel means of population readout: The property that is read out, symmetry, is not a property to which the population units are sensitive. The method uses all the information in the KA and offers a way of solving the correspondence problem in binocular vision that can be applied directly to motion, as discussed below.

We demonstrated the use of false-match symmetry for detecting surfaces camouflaged within RDSs. False matches dominate the correspondences between pairs of these images, making the identification of true matches a challenge. Biological stereo systems can in many instances solve the RDS correspondence problem readily, even in the presence of considerable between-image distortion [12]. This is despite the fact that objects rendered in depth in RDSs are defined solely by the correlation between the L and R stereo images. Because the target objects are not contained in either L or R image alone, correspondence performance is not limited by within-image object segmentation requirements. Hence, RDSs are an excellent format for testing correspondence algorithms. Compared with conventional images, RDSs also magnify the number of false matches, which serves our present purpose.

The correspondence problem can be resolved directly, by selecting true matches, or indirectly, by using the flanking patterns of false matches to locate the true matches. The sought-after image properties in the two cases are quite different. The false-match parameters, unlike the true-match parameters, are not those associated with the objects that produced the images. Instead, they are properties of associated with symmetrical regions of false matches and the transformations operating on these regions. Frontoparallel surfaces, slanted planar surfaces, and curved surfaces, when covered with non-unique texture elements, create distinct transformations of false-match symmetry in the KA. In the orthogonal KAs used here, true matches of frontoparallel surfaces are flanked by regions of false matches with reflection symmetry. True matches for slanted surfaces are flanked by symmetrical reflections of false-matches that are scaled by a constant (Fig 6). The symmetry of false matches from curved surfaces have systematically varying aspect ratios.

A frontoparallel surface extending across the visual field along the horopter divides the KA into reflected positive and negative disparity domains. Tyler [8,9] suggested disparity averaging along lines of sight as a means of cancelling the false-match disparity values, thereby revealing the presence of the true matches. For other cases, including off-horopter frontoparallel surfaces and slanted or curved surfaces, line-of-sight averaging does not lead to cancellation. However, false-match symmetry does not have to fill the entire KA or fall along lines of sight in order to be useful. Characterizing the transformation that maps the relatively ‘near’ false matches onto the relatively ‘far’ false matches gives the disparity profile of the true-match candidates lying on the axis of symmetry that divides them.

Using example frontoparallel, slanted, and curved surfaces rendered by the correlation between L and R random-dot images, we showed the feasibility of selecting true matches by examining the flanking false-match regions. Our demonstration was restricted to a horizontal dimension. Even so, the algorithm was relatively computationally intensive given the image size. For purposes of demonstration, this is not of great concern; efficiency was not our goal. For other purposes, the desired resolution could vary with the expected parameters of target surfaces. Other computational strategies exist. Graphical methods dedicated to the detection of symmetry, for example, are not considered here but would be of obvious utility.

#### Comparison with other approaches

The goal of solving the correspondence problem is to find likely true matches. Our method differs from others in that it approaches this goal indirectly. Instead of searching for matches with the properties expected of true matches, it searches for false matches having properties that lead to the location of true matches. These false-match properties are not the properties of likely true matches—smoothness, for example. Instead, the sought-after property is a relationship between regions of false matches.

Though this is a novel approach, the algorithm we use to find symmetry has features in common with other biologically inspired stereo-correspondence algorithms. Even so, the commonalities are partial and come with critical antitheses. Many traditional correspondence algorithms are dense matching processes in that they employ spatial filtering at various scales and positions to identify corresponding points or cooperative processes involving networks of excitatory and inhibitory elements. For example, Marr and Poggio’s 1979 [3] algorithm detects true matches consistent with smoothness by spatially filtering and detecting correspondence first at a coarse level, which are then used as guides to bring finer details into correspondence. The disparity map is built up sequentially. Such multi-resolution filtering is a common way to generate a disparity space and it is used in our algorithm.

In algorithms like this, true matches are sought within disparity ranges and gradients that enforce a smoothness constraint. Smoothness is supported with a range around the x-axis (or the x-y plane) and is inhibited along the z-axis (e.g., [2,13]). In seeking false-match symmetry, our approach looks in the opposite direction. Symmetry is sought along an axis offset from that the surface—normal to the surface in the case of frontoparallel surfaces (in general, angled from the frontoparallel normal by -*θ* for a surface with slant *θ*). In addition, and in contrast to many other approaches, it requires no edge or zero-crossing detection, no feature identification, no image segmentation, and no constraints to the range of disparities or temporal delay in detecting fine ones. Because the signals it exploits are complementary to those used elsewhere, our algorithm can be combined readily with others. This could produce a flexible system of depth and surface profile detection suitable also for motion-in-depth analysis.

Motion, like binocularity, has a correspondence problem: finding true matches among false ones in images separated in time rather than in space. This makes the KA’s false-match symmetry useful for jointly extracting stereo depth and motion signals. Extending the false-match symmetry analysis to motion is made easy by relabeling: Changing the ‘Left/Right Retina’ axis labels in Figs 1–4 to ‘Single Retina at Time 1/Time 2’ gives a coding of 2-frame monocular object motion (extendable to ‘Time 2/Time 3’ and so on.). Bilateral (top/bottom) mirror-image symmetry—hence, true matches confined to the KA’s horizontal bisector, from corner to corner—represents a static display, the motion analog of a frontoparallel plane with zero disparity.

At the more peripheral level of binocular correlation, our implementation again employs elements found in other models. Its use of binocular summation and rectification, for example, is seemingly similar to that found in the disparity energy model [14,15]. However, the energy model computes correlation by employing units that vary in either the position or phase of the receptive fields in quadrature. Its purpose, like that of other binocular matching mechanisms, is to detect correspondences between images, not to distinguish true and false correspondences. Hence, its output, over an array of units, is a KA, complete with the correspondence problem. By contrast, our approach uses odd-symmetric kernels in antiphase throughout to ensure detection of false-match symmetry and to solve the correspondence problem. True matches are scored lowly and disregarded. Unlike the energy model’s output, signals in our KAs are not depth readouts. Computed signals in the KAs indicate the confidence of the presence of symmetry. The position within the KA where this signal is found to be consistent across kernel sizes corresponds to the depth profile. Most fundamentally, other approaches differ from false-match symmetry in that they function best where our approach would fail: in images that lack false matches.

**Fig 11:**
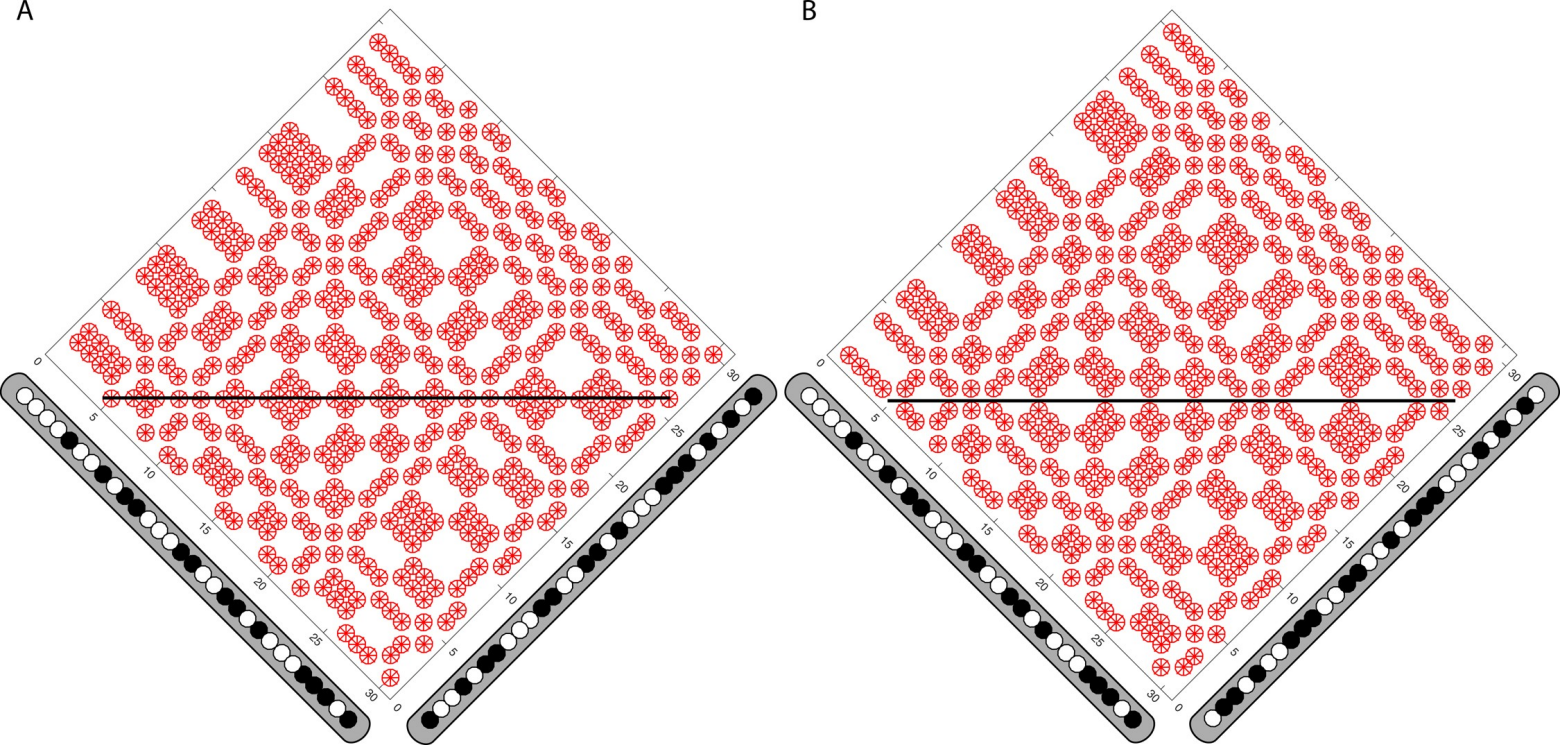
Effect of anti-correlation. (A) The KA associated with correlated random-dot input. Binary L and R input is in the margins. Matching points in the KA are marked in red. The horizontal line runs along the axis of symmetry, cutting through the true matches for a frontoparallel surface. (B) The inverted KA associated with the anti-correlated version of the input seen in (A). The axis of symmetry is unchanged, but now passes through mismatches.

The disparity energy model has been quite successful in accounting for behavioral and physiological responses to disparity. It predicts disparity-selective responses not only to correlated input, but also to anti-correlated input. Anti-correlated stimuli produce a sign-reversed response in disparity energy units, a response seen in neurons in several areas of primate visual cortex [9,10,16], though without an accompanying perception of depth of densely textured surfaces [17–19]. The Keplerian response to anti-correlated input is the inverse of the matching array produced by the correlated version of the same input. Matches and mismatches in the KA switch places, preserving the symmetry parameters (Fig 11). It is clear that false-match symmetry, if used in biological systems, would locate true matches rather than produce perceptions of depth directly; since only mismatches would be found along the symmetry axis, observers would not see depth from anti-correlated stereograms. It is also clear that the presence of true matches is not necessary for false-match symmetry to locate the symmetry axis; false matches locate the same symmetry axis whether the input is correlated or anti-correlated, though true matches (as usually defined) exist in one and not the other. The perception of reversed depth in anticorrelated input, to the extent it occurs at all, is found primarily in sparse visual patterns, such as isolated lines or dots [20]. Such situations eliminate mismatches, perhaps allowing the output of local correlators, rather than matches along a symmetry axis, to drive perception.

### Further biological considerations

Many low-level binocular neurons in mammals, whose receptive fields span a range of spatial frequencies, orientations, and sizes, also code disparities between the eyes [21]. Individual neurons are tuned to a limited range of disparity values typically related to receptive field size (the ‘size-disparity correlation’) [22–24]. This is sufficient for the ensemble of neural responses to disparity to form a Keplerian array. But the KAs like those shown in Figs 1–4 are fully connected and code all disparities regardless of size, whereas biology favors local connections and limited means of coding large disparities. The maximum disparity that yields perceived depth varies with stimulus scale and retinal eccentricity, but in humans it may be as large as 10° of visual angle or more (reviewed in [21]), far exceeding the fusible range. Even so, a biologically instantiated KA of sufficient lateral extent would lack representation of very large disparities. A disparity limit would affect the upper and lower apices of KAs like those shown in Fig 1, where disparities are largest.

A measure of whether the correspondence problem has actually been solved in a particular cortical area can be gained by examining neural responses to false matches. Physiological recordings in primate inferior temporal cortex, reveals a dorsal (or ‘what’) pathway in which neurons respond to correlated RDS displays of object surfaces but not to anticorrelated RDS’s [25]. This provides evidence of a selective response to true matches and a rejection of false matches at or before activation of this region of cortex. But multiple areas along the dorsal (‘where’) pathway do respond well to anticorrelated stereograms [16], indicating that the correspondence problem has not been solved. These dorsal areas are also strongly tuned to motion. Thus, false matches appear to be propagated through a series of functional areas that might be used in the computation of motion in depth. The transformations that relate the symmetrical blocks of false matches that flank true match also characterize solutions to the motion correspondence problem for rigid object surfaces moving in three-dimensional space [26,27]. Hence, the coding of false-match structure may be isomorphic with the coding of motion-defined spatial structure and both may be analyzed by the same circuitry and computational strategy [28–30]. Supplementing evidence of use of false-match symmetry for depth perception [31] with moving stimuli would provide a means of testing the computation behind perceived motion in depth.

**Fig 12:**
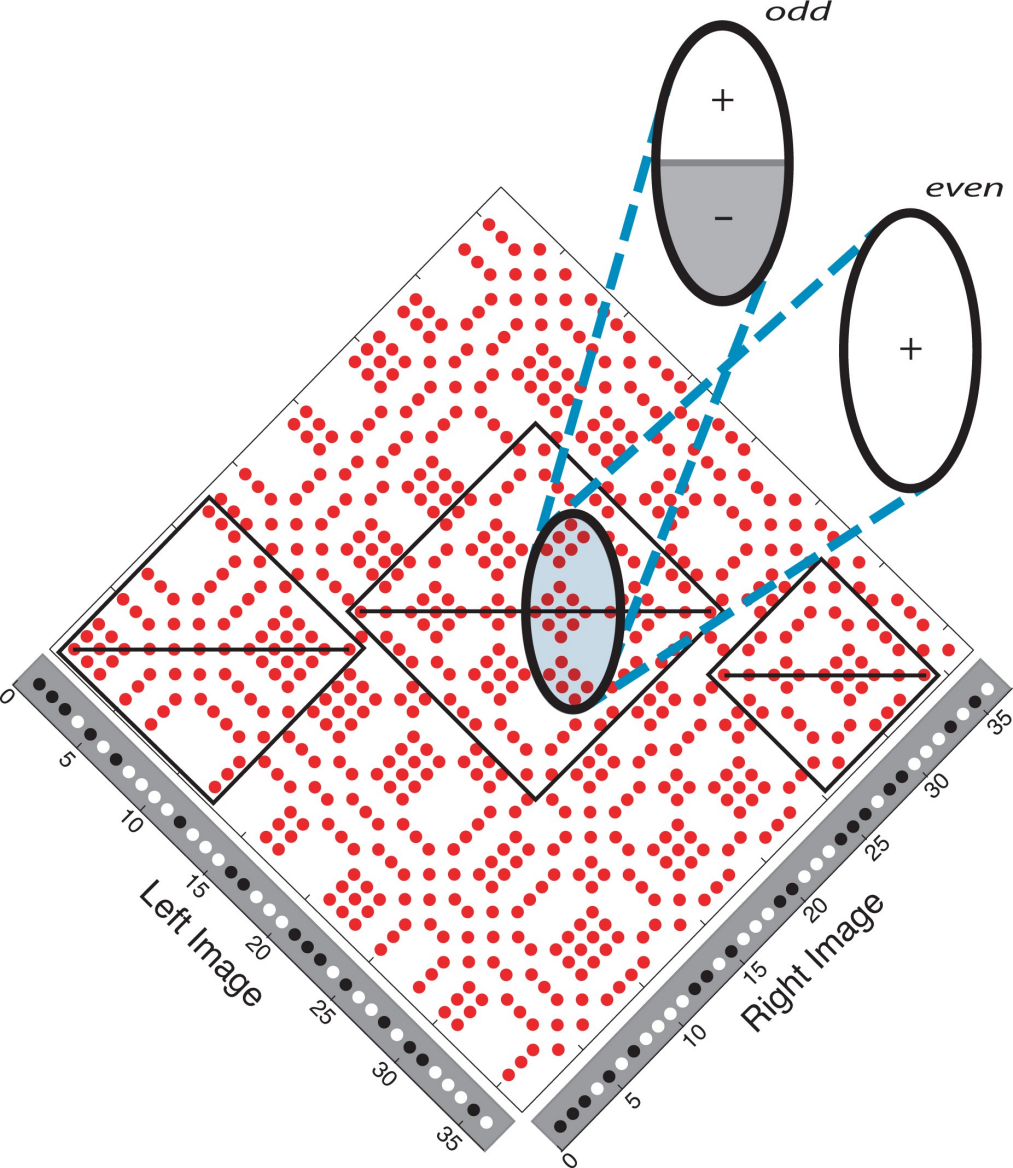
Keplerian array overlaid with a co-extensive pair of receptive fields. The receptive field, one with even symmetry and one with odd symmetry, straddle a row of true matches. In this position, the odd receptive field, if no larger than the false-match symmetry zone, will give an output of zero. The even receptive field, if not summing over mismatches only, will give a non-zero output. The ratio of the outputs over the two receptive fields serves as a symmetry detector.

Symmetry detection, too, can be and has been implemented biologically, as shown by ample data starting with Mach’s [32] observations on the human response to spatially symmetrical patterns. Our detection problem is similar; instead of detecting a spatially symmetrical pattern of visual input, as concerned Mach, we have the problem of detecting a pattern of neural responses to symmetrical relative disparity values. An easily visualized and biologically realistic version of our algorithm relies on the Fourier transform of a symmetrical signal being a real function, the sine terms having a coefficient of zero. It compares the responses of two neurons with co-located receptive fields (filters), one with even symmetry and one with odd symmetry—counterparts of the transform’s cosine and sine terms. This is shown in Fig 12, where the receptive field pair is aligned with a row of true matches. High values of the even:odd output ratio would identify candidate regions of reflection symmetry. An odd receptive field centered on the symmetry axis will output a low (ideally, zero) value; the co-located even receptive field will output a low value of integrated nodes only if those nodes are mismatches (outputting zero). The even receptive field thus serves a control function: It negates the effect of odd receptive fields whose low output is due to input from mismatches rather than symmetrically distributed matches. This, like the basic false-match symmetry concept, is entirely a post-matching computation, so any correlator—energy-model neurons, for example—could be used to generate the KA in which symmetry is detected. False-match symmetry, in other words, operates in the realm of global rather than local stereopsis [1].

In order to detect surfaces of different extents and slants, families of receptive field pairs, spanning a range of sizes and orientations, would have to tile the KA. Still, curves surfaces would be difficult to detect by this method, unless they were approximated by slanted segments and integrated into a coherent surface at a later stage of processing. Nevertheless, the neural components and the required computations are familiar ones that functionally recapitulate the algorithm we used here.

## Supporting information

S1 Text Supplementary methods. (PDF)

S1 Fig The algorithm’s procedure illustrated in 6 steps. (PDF)

S2 Fig Ground truth. Ground truth (ideal solutions) appear in (A) for horopter stimulus (for results shown in Fig 7), (B) for frontoparallel stimulus (for results shown in Fig 8A), (C) for 26.5° backward slant (for results shown in Fig 8B), (D & E) for backward and forward slants (for results shown in Fig 9), and (F) for noisy frontoparalel stimuli (for results shown in Figs 10 and S3).
(PDF)

S3 Fig Effect of noise. Solutions obtained when (A) pixel intensity noise varied between 0% and 30%, and (B) the proportion of pixels with 5% noise rose from 25% to 100%. SNRs are graphed in Fig 10.
(PDF)

## References

[1] Julesz B. Global Stereopsis: Cooperative phenomena in stereoscopic depth perception In: Held R, Leibowitz H, Teuber H (Eds.) Handbook of Sensory Physiology, v. 8, Perception. Springer, Berlin, Heidelberg, 1978; pp. 215–256.

[2] Marr D, Poggio T. Cooperative computation of disparity. Science. 1976; 194:283–287. 10.1126/science.968482 968482

[3] Marr D, Poggio T. A computational theory of human stereo vision. Proc. R. Soc. Lond. B. 1979; 204:301–328. 10.1098/rspb.1979.0029 37518

[4] Ma J, Zhao J, Tian J, Yuille A, Tu Z. Robust point matching via vector field consensus. IEEE Trans Image Process. 2014; 23(4): 1706–1721. 10.1109/TIP.2014.2307478 24808341 PMC5748387

[5] Liu J, Li C, Mei F, Wang Z. 3D entity-based stereo matching with ground control points and joint second-order smoothness prior. Vis. Comput. 2015; 31:1253–1269. 10.1007/s00371-014-1009-3

[6] Zhu Z, Dai Q. Hybrid scheme for accurate stereo matching. Neurocomputing. 2017; 252: 24–33. 10.1016/j.neucom.2016.11.0835

[7] Scharstein D, Szeliski R. A taxonomy and evaluation of dense two-frame stereo correspondence algorithms. Intl J Comp Vis. 2002; 47:7–42.

[8] Tyler C. Stereomovement from interocular delay in dynamic visual noise: A random spatial disparity hypothesis Amer J Opt. 1977; 54:374–386. 10.1097/00006324-197706000-00005 907026

[9] Tyler C. Cyclopean vision In: Vision and Visual Dysfunction, v. 9, Binocular Vision. Regan D. (Ed.), MacMillan: New York, 1991; pp. 38–74.

[10] Cumming B, DeAngelis G. The physiology of stereopsis, Ann Rev Neurosci. 2001; 24:203–238. 10.1146/annurev.neuro.24.1.203 11283310

[11] Hibbard P, Scott-Brown K, Haigh E, Adrain M. Depth perception not found in human observers for static or dynamic anti-correlated random dot stereograms PLOS One. 2014; 9:1–9. 10.1371/journal.pone.0084087 PMC3885516 24416195

[12] Julesz B. Foundations of Cyclopean Perception. Univ. of Chicago Press, Chicago, 1971.

[13] Zitnick C, Kanade T. A cooperative algorithm for stereo matching and occlusion detection. IEEE Trans. Pat. Anal. Mach. Intell. 2000; 22:675–684.

[14] Ohzawa I, DeAngelis G, Freeman R. Stereoscopic depth discrimination in the visual cortex: neurons ideally suited as disparity detectors. Science, 1990; 249:1037–1041. 10.1126/science.2396096 2396096

[15] Qian N, Zhu Y. Physiological computation of binocular disparity. Vis. Res., 1997; 37:1811–1827. 9274767 10.1016/s0042-6989(96)00331-8

[16] Cumming B, Parker A. Responses of primary vision cortical neurons to binocular disparity without depth perception. Nature. 1997; 389:280–283. 10.1038/38487 9305841

[17] Cogan A, Lomakin A, Rossi A. Depth in anticorrelated stereograms: effects of spatial density and interocular delay. Vis. Res., 1993; 33:1959–1975. 8249313 10.1016/0042-6989(93)90021-n

[18] Cumming B, Shapiro S, Parker A. Disparity detection in anticorrelated stereograms. Percept., 1998; 27:1367–1377. 10.1068/p271367 10505181

[19] Hibbard P, Scott-Brown K, Haigh E, Adrain M. Depth perception not found in human observers for static or dynamic anti-correlated random dot stereograms. PLoS ONE, 2014; 9:1–9. e84087. 10.1371/journal.pone.0084087 24416195 PMC3885516

[20] Julesz B. Binocular depth perception of computer-generated patterns. Bell Labs Tech. J., 1960; 39:1125–1162.

[21] Howard I, Rogers B. Binocular Vision and Stereopsis, v. 2, New York/Oxford: Oxford University Press/ Clarendon Press, 1995

[22] Felton T, Richards W, Smith R. Disparity processing of spatial frequencies in man. J. Physiol. (Lond.). 1972; 225: 349–362. 5074392 10.1113/jphysiol.1972.sp009944 PMC1331110

[23] Prince S, Cumming B, Parker A. Range and mechanism of encoding of horizontal disparity in macaque V1. J Neurophysiol. 2002; 87: 209–221. 10.1152/jn.00466.2000 11784743

[24] Smallman H, MacLeod D. Size-disparity correlation in stereopsis at contrast threshold. J Opt Soc Amer. 1994; 11: 2169–2183. 10.1364/josaa.11.002169 7931758

[25] Janssen P, Vogels R, Liu Y, Orban G. At least at the level of inferior temporal cortex, the stereo correspondence problem is solved, Neuron. 2003; 37:693–701. 12597865 10.1016/s0896-6273(03)00023-0

[26] Koenderink J, van Doorn A. Geometry of binocular vision and a model for stereopsis, Cybern. 1976; 21:29–35. 10.1007/BF00326670 1244864

[27] Koenderink J, van Doorn A. Affine structure from motion, J Opt Soc Amer A. 1991; 8:377–385. 2007912 10.1364/josaa.8.000377

[28] Fernandez J, Watson B, Qian N. Computing relief structure from motion with a distributed velocity and disparity representation. Vis Res. 2002; 42:883–898. 11927353 10.1016/s0042-6989(02)00023-8

[29] Fernandez J, Farell B. A new theory of structure-from-motion perception, J Vis. 2009; 9:1–20. 10.1167/9.11.23 PMC5634789 20053086

[30] Fernandez J, Farell B. A neural model for the integration of stereopsis and motion parallax in structure from motion. Neurocomput. 2008; 71:1629–1641. 10.1016/j.neucom.2007.04.006 PMC2597843 19255615

[31] Farell B. Solving the binocular correspondence problem with ghost matches. J Vis. 2013; 13:933.

[32] Mach E. The Analysis of Sensations. (C Williams, Trans.). Dover Edition: New York, 1887/1959.

