# Correction: Solving the stereo correspondence problem with false matches

**DOI:** 10.1371/journal.pone.0221478

**Published:** 2019-08-15

**Authors:** The PLOS ONE Staff

Due to a typesetting error, the Data Availability statement for this paper is incorrect. The publisher apologizes for the error. The correct statement is: All relevant data are available at DOI: 10.5281/zenodo.3334347.
