# Supplementary material for: Solving the stereo correspondence problem with false matches
Source: PLoS One. 2019 Jul 29;14(7):e0219052. doi: 10.1371/journal.pone.0219052 (PMC6662999; doi:10.1371/journal.pone.0219052)
Supplement: S1 Text — (PDF) [file pone.0219052.s001.pdf]

Solving the stereo correspondence problem with false matches

Cherlyn J. Ng, Bart Farell
Syracuse University

Supplementary information

<u>Supplementary Methods</u>

Image processing

Left and right images were filtered (convolved) with odd-symmetric Gabor kernels in anti-phase. The kernels came in different sizes. Examples of a pair of odd symmetric Gabor kernels of the same size placed on the left and right images are shown in the marginals of Figure A. Because the left kernel was in antiphase of the right, binocular summation would be equivalent to filtering by an odd-symmetric binocular receptive field (major panel; Figure A).

Symmetrical false matches would produce identical by oppositely signed convolution products in the two halves of the odd–symmetric binocular receptive field. Combining these two halves results in a null response. Elsewhere, products without symmetry would not nullify across the two-halves of the receptive field. Thus, regions of nullification indicate the possible presence of symmetry. However, a null result could also have been produced from an absence of false matches. To circumvent this issue, we rectified the KA (through squaring) before integrating the binocular receptive field (Figure B). Rectification produced non-zero outcomes and boosts evidence for false-match symmetry.

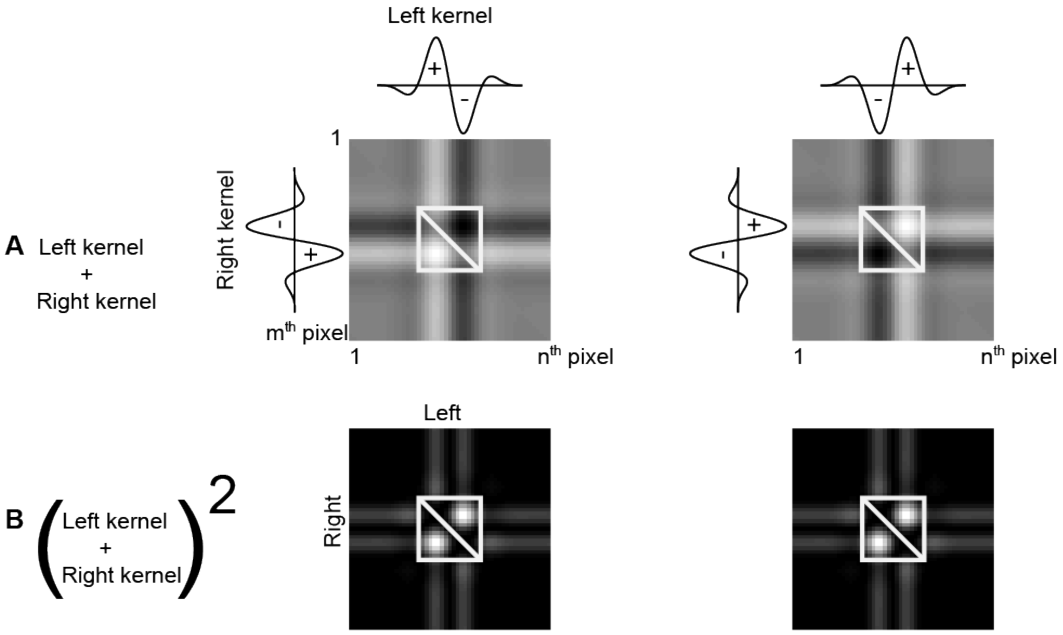

Figure A. Binocular receptive fields (A) before and (B) after rectification. (A) Two instances of left and right kernels in antiphase. Summation results in odd-symmetric binocular receptive fields in which balanced negative (dark) and positive (light) regions lie within a symmetrical zone (white box) on either side of true matches along the symmetry axis (white diagonal). Cancellation—near zero outcomes—resulting from integrating over these regions is consistent with the presence of false-match symmetry. (B) Rectification of (A). Rectification would give positive outcomes. The two antiphase instances in (A) would now give identical results (left and right panels) that can be combined to improve redundancy by two-fold.

Convolution at multiple scales to accumulate evidence

The optimum binocular receptive field, and hence its component Gabor filters, depends on the properties of the true-match depth plane. For instance, slanted planes require uneven L and R kernels; leftward slants require smaller kernels in the left image than in the right (Figure B left panel), while rightward slants are opposite (right panel). Similarly, small true-match depth planes produce small regions of false match symmetry that are well detected by small kernels, but large planes need large kernels in addition to capture the entire symmetrical region. An important procedural question

to address is this: When no information is provided about the ground truth (the case in our simulations), how should kernels be applied in order to optimally detect symmetry?

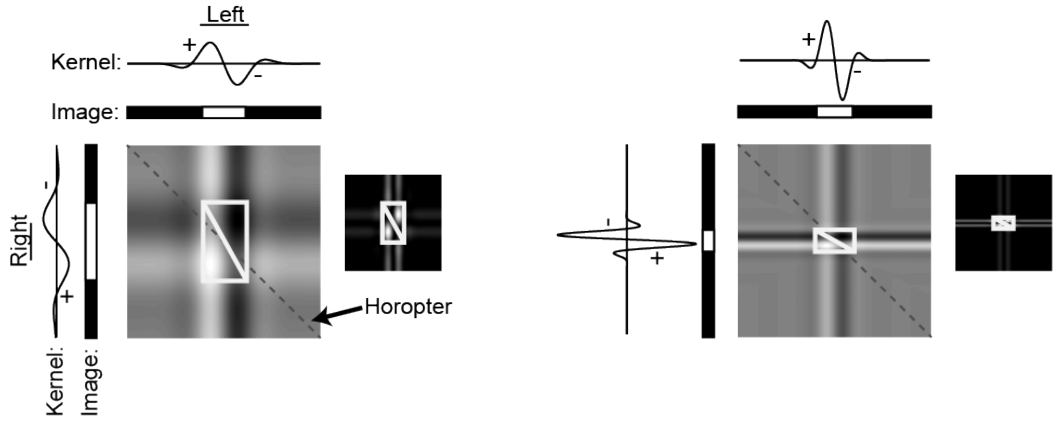

Figure B. The aspect ratio of the optimum binocular receptive field reflects surface slant, which gives matching L and R regions their relative sizes. Two examples are presented here: a large surface (white line) that slants forward of the horopter (dashed gray line), and a small surface that slants backwards. Accordingly, the projection onto the left and right images differ as do the symmetrical region around the surfaces (white boxes) and the optimum set of kernels needed to detect symmetry. Insets show the rectified binocular receptive fields.

To ensure detectability of false match symmetry at every scale and L:R aspect ratio, we first convolved the left and right images with kernels of all sizes ranging from the Nyquist limit to whole images. Then, we combined the left convolution products of every kernel size with the right convolution products of every kernel size. If symmetry was detected in a particular Keplerian array, sizes of its corresponding L and R kernels were noted. Then, evidence of symmetry would be sought in other Keplerian arrays with the same L:R kernel aspect ratios. If false-match symmetry were in fact present, other members of the same aspect-ratio family would also

possess symmetry. Evidence was accumulated within that particular aspect-ratio family up to kernels of a size beyond which evidence for symmetry breaks down (Figure C). This maximum kernel size corresponds with extent of the candidate symmetry axis.

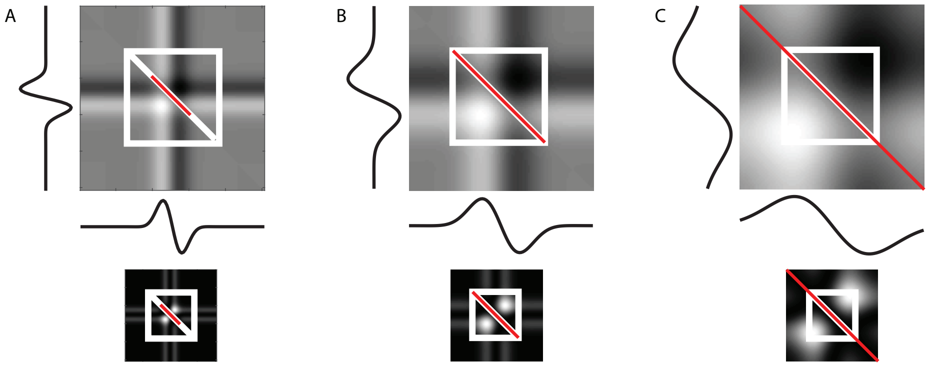

Figure C. (A) An instance of small kernels that capture part (red diagonal) of a symmetry region (white box) around the true matches (white diagonal). (B) Kernel sizes that correspond to the entire region of symmetry and no more. (C) Kernels too big for the region of symmetry, incorporating noise from non-symmetrical regions at the outskirts. The algorithm works by finding the smallest bandwidth that contains symmetric false matches (A), and then searching KAs with larger bandwidths and the same aspect ratio (B), up to the size at which symmetry is no longer detected (C).

Thresholding and match selection

Kernels of different sizes can give incommensurate outputs to equally informative input; for example, a big receptive field sensing a small white dot against a black background would give an impoverished response as compared to a small RF sensing the same dot. In order to combine evidence for L-R matches across kernels of different sizes, we simulated a simple gain control mechanism to scale and normalize the KAs from the procedure above. The KAs obtained from the convolution and summation process were grouped according to their binocular bandwidth. Binocular bandwidth corresponds to the area in a KA covered by the L X R kernels (Figure B,

white boxes). By this definition, inspired by [1], any combination of L and R kernels that covers the same area has the same bandwidth. The assumption is that image-processing outcomes from kernels of the same bandwidth sampled the environment similarly and hence can be normalized, scaled and analyzed together.

We normalized and scaled KAs of the same bandwidth. Pixelwise signals within KAs of the same binocular bandwidth were binned. A cumulative histogram of these binned signals was fit with a cumulative Weibull model. Then the decorrelated scores predicted by the Weibull model fit replaced empirical outcomes at each binocular pixel of the corresponding KAs.

After these gain-control steps, we ranked the evidence for the symmetry of matches within each aspect-ratio family. We set a threshold percentile on the fitted Weibull model such that any score above this threshold was regarded as evidence for symmetry and kept, while values below the threshold were suppressed. In all simulations without noise, threshold was 0.0001 from the maximum score of 1 (that is, the threshold was the 1-0.0001th percentile rank). The threshold was relaxed to 0.0002 from the maximum score when intensity noise was above 10%. It is important to note here that these decorrelated and thresholded scores within KAs are not depth readouts. Instead, they indicate the confidence for symmetry at a particular location in the KA.

Supplementary Figure D. The algorithm's procedure in six steps.

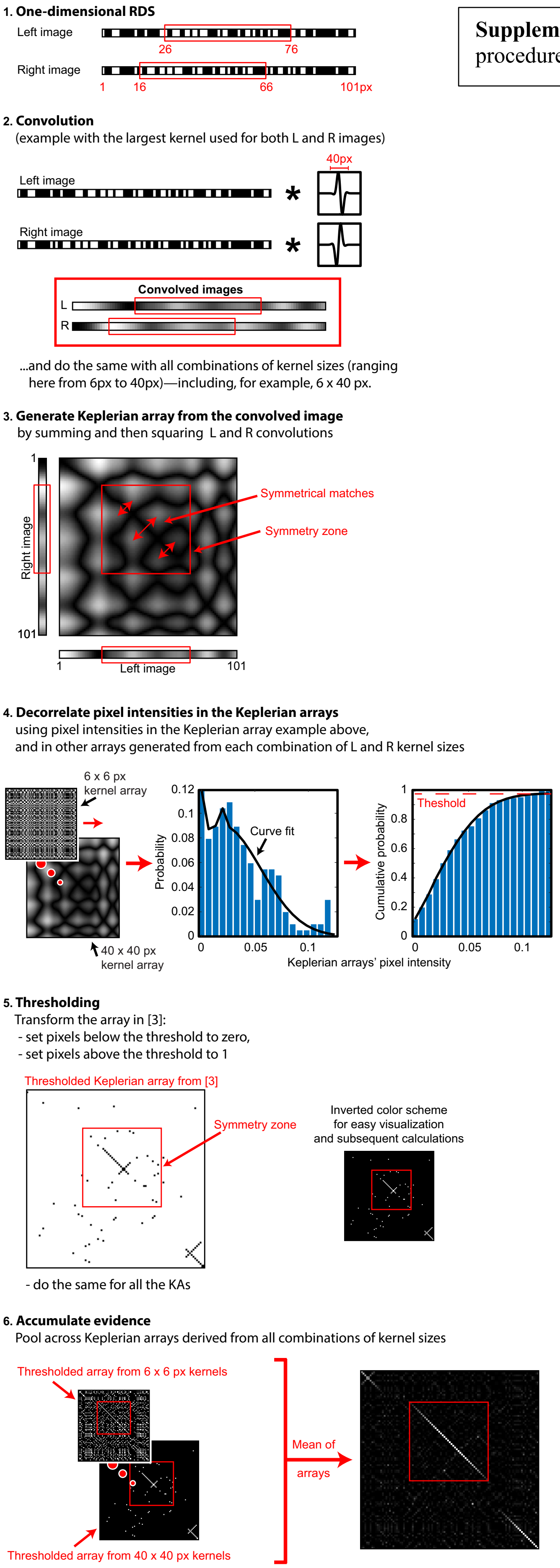

Supplementary Results

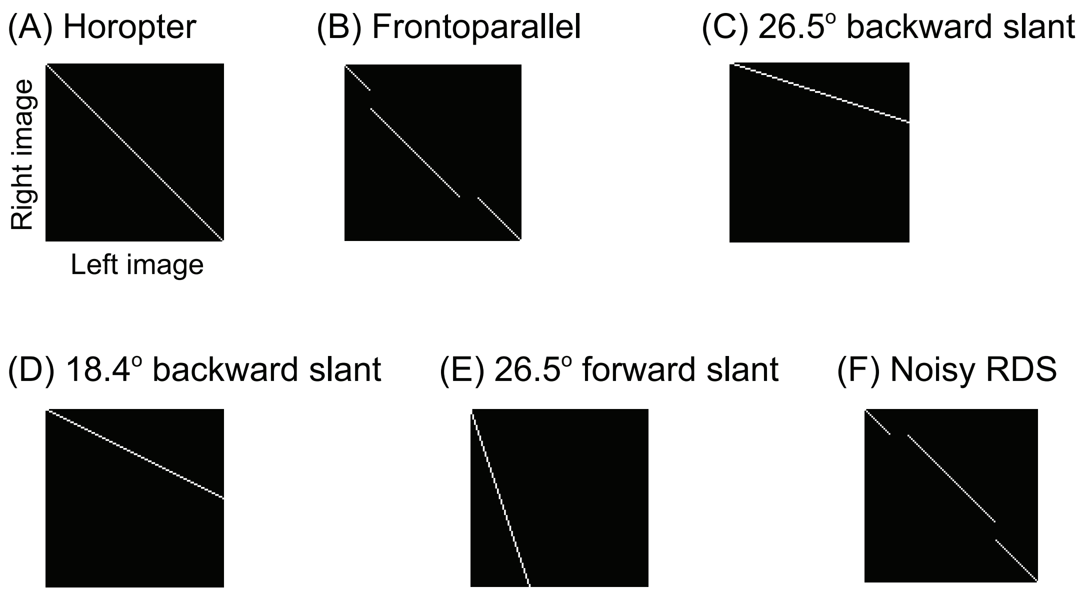

Figure E. Ground truth (ideal solutions) to (A) horopter: Figure 7, (B) frontoparallel: Figure 8A, (C) 26.5° backward slant: Figure 8B, (D & E) backward and forward slants of Figure 9 and (F) noisy frontoparallel RDS stimuli: Figure 10 and Figure F.

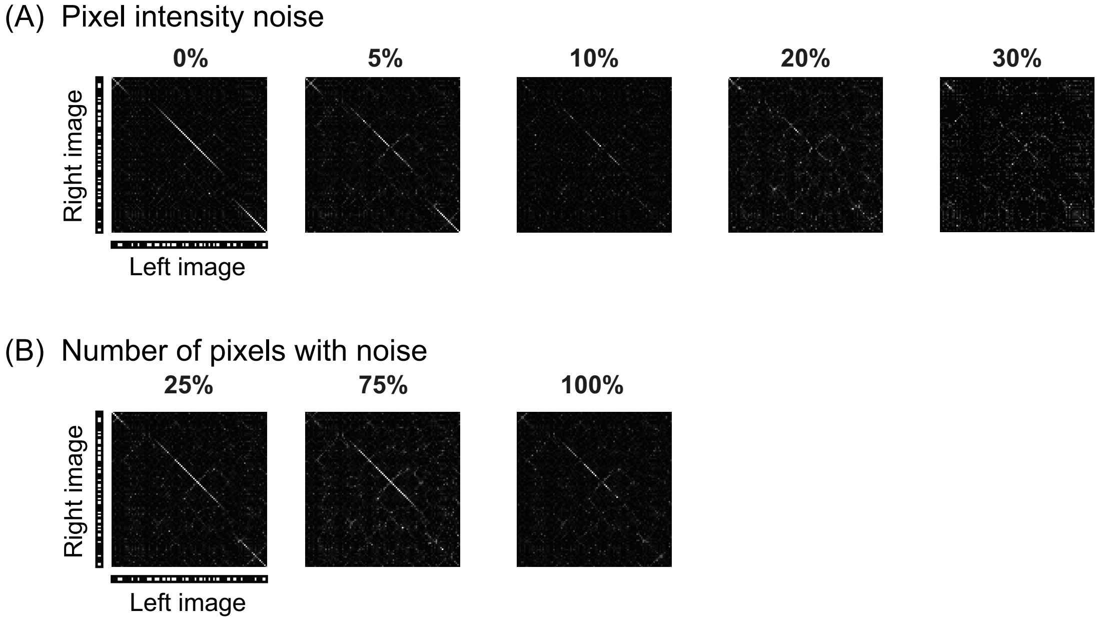

Figure F. Solutions obtained when (A) pixel intensity noise varied between 0% to 30%, and (B) the number of pixels with 5% intensity noise rose from 25% to 100%. SNR are graphed in Figure 10.

References

[1] Gabor D. Theory of communication. Part 1: the analysis of information. *J IEE* (London). 1946; 93:429-457.
