# Supplementary material for: Solving the stereo correspondence problem with false matches
Source: PLoS One. 2019 Jul 29;14(7):e0219052. doi: 10.1371/journal.pone.0219052 (PMC6662999; doi:10.1371/journal.pone.0219052)

1. One-dimensional RDS

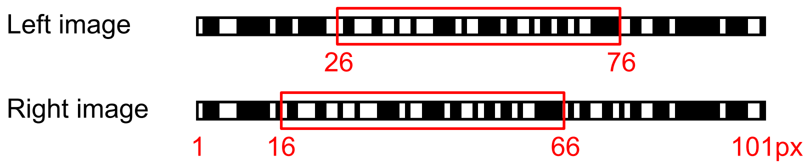

2. Convolution
(example with the largest kernel used for both L and R images)

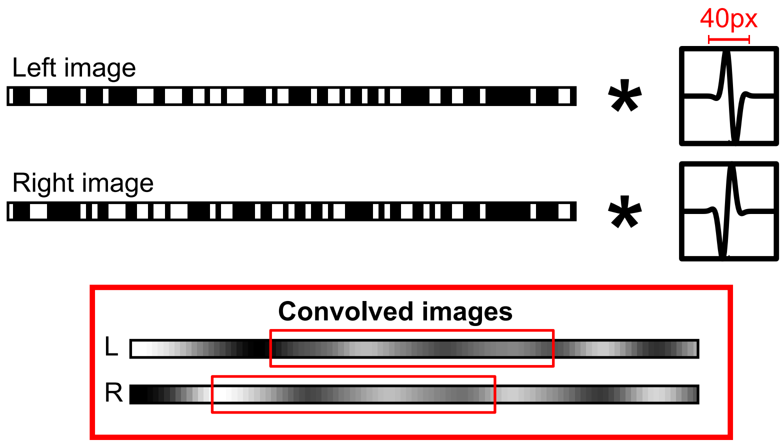

...and do the same with all combinations of kernel sizes (ranging here from 6px to 40px)—including, for example, 6 x 40 px.

3. Generate Keplerian array from the convolved image
by summing and then squaring L and R convolutions

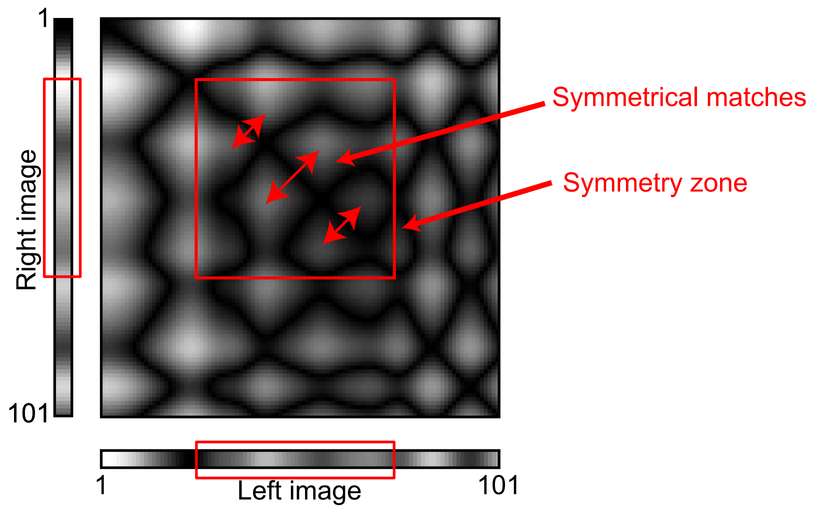

4. Decorrelate pixel intensities in the Keplerian arrays
using pixel intensities in the Keplerian array example above,
and in other arrays generated from each combination of L and R kernel sizes

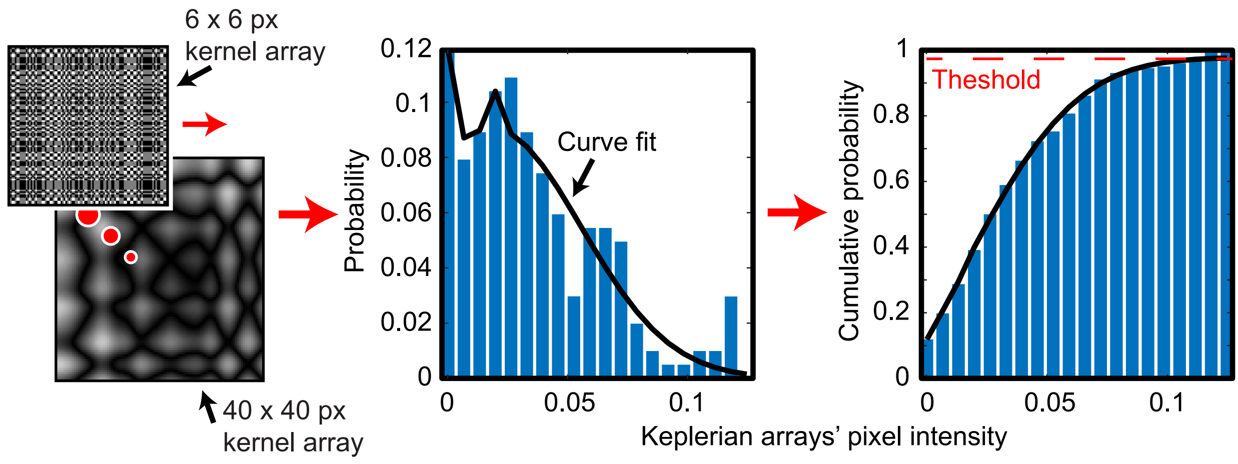

5. Thresholding
Transform the array in [3]:
- set pixels below the threshold to zero,
- set pixels above the threshold to 1

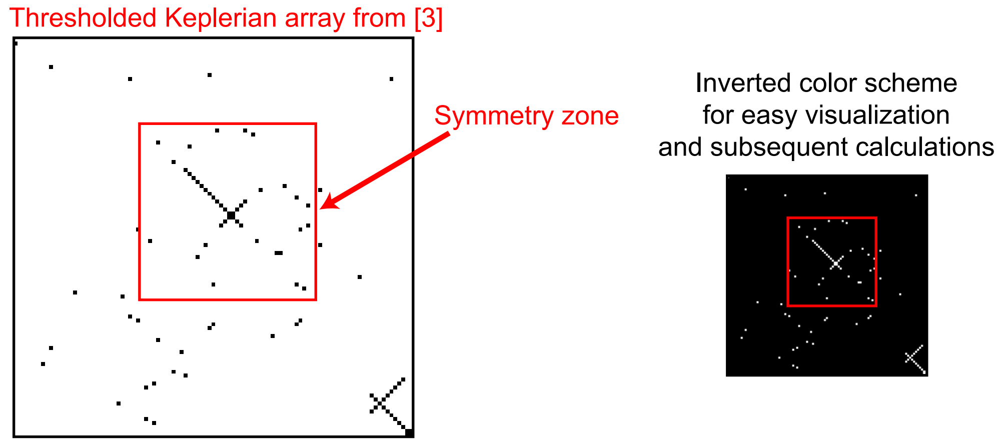

- do the same for all the KAs

6. Accumulate evidence
Pool across Keplerian arrays derived from all combinations of kernel sizes

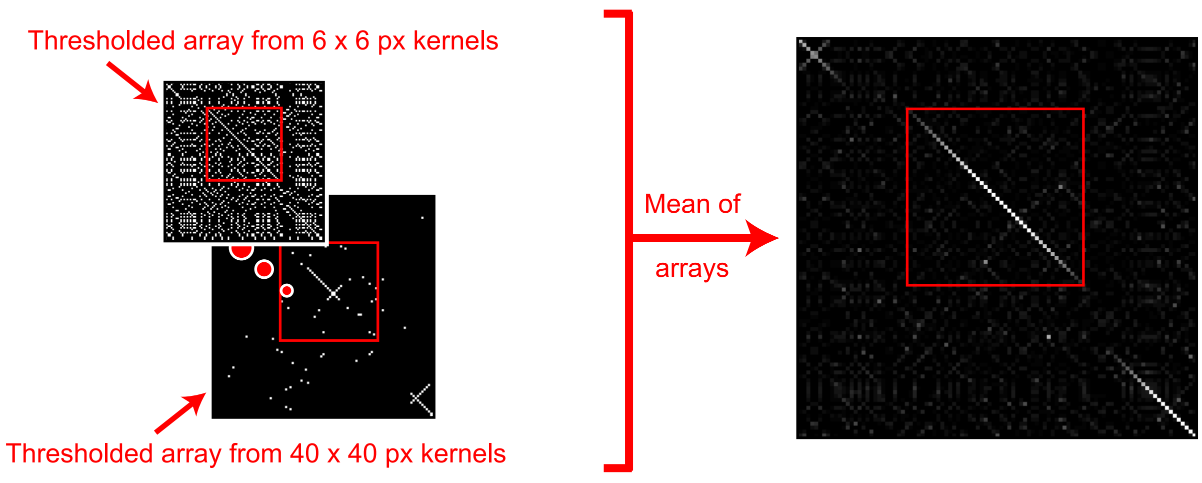

Supplement: S1 Fig — (PDF) [file pone.0219052.s002.pdf]
