# Supplementary material for: Solving the stereo correspondence problem with false matches
Source: PLoS One. 2019 Jul 29;14(7):e0219052. doi: 10.1371/journal.pone.0219052 (PMC6662999; doi:10.1371/journal.pone.0219052)
Supplement: S2 Fig — Ground truth (ideal solutions) appear in (A) for horopter stimulus (for results shown in Fig 7), (B) for frontoparallel stimulus (for results shown in Fig 8A), (C) for 26.5° backward slant (for results shown in Fig 8B), (D & E) for backward and forward slants (for results shown in Fig 9), and (F) for noisy frontoparalel stimuli (for results shown in Figs 10 and S3). (PDF) [file pone.0219052.s003.pdf]

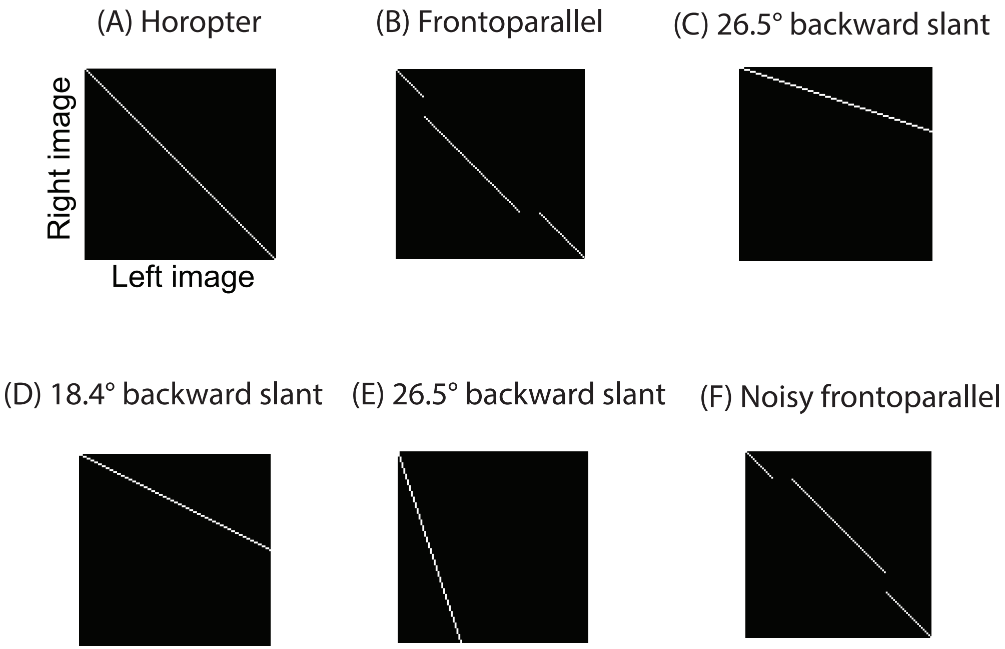
(A) Horopter
(B) Frontoparallel
(C) 26.5° backward slant
Right image
Left image
(D) 18.4° backward slant
(E) 26.5° backward slant
(F) Noisy frontoparallel
