# Supplementary figures and images for: Solving the stereo correspondence problem with false matches
Source: PLoS One. 2019 Jul 29;14(7):e0219052. doi: 10.1371/journal.pone.0219052 (PMC6662999; doi:10.1371/journal.pone.0219052)

(A) Pixel intensity noise

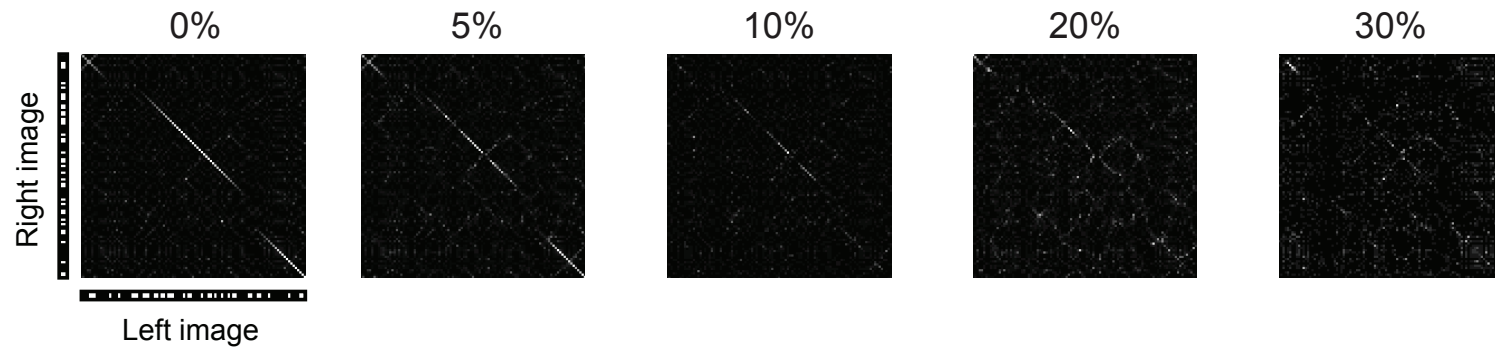

(B) Proportion of pixels with noise

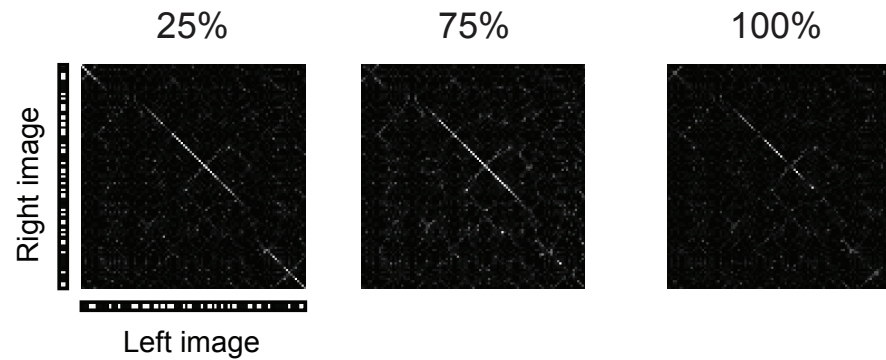

Supplement: S3 Fig — Solutions obtained when (A) pixel intensity noise varied between 0% and 30%, and (B) the proportion of pixels with 5% noise rose from 25% to 100%. SNRs are graphed in Fig 10. (PDF) [file pone.0219052.s004.pdf]
